# Genome-wide regulation of electro-acupuncture on the neural *Stat*5-loss-induced obese mice

**DOI:** 10.1371/journal.pone.0181948

**Published:** 2017-08-14

**Authors:** Shu-Ping Fu, Hao Hong, Sheng-Feng Lu, Chen-Jun Hu, Hou-Xi Xu, Qian Li, Mei-Ling Yu, Chen Ou, Jian-Zhong Meng, Tian-Lin Wang, Lothar Hennighausen, Bing-Mei Zhu

**Affiliations:** 1 Key Laboratory of Acupuncture and Medicine Research of Ministry of Education, Nanjing University of Chinese Medicine, Nanjing, China; 2 School of Information Technology, Nanjing University of Chinese Medicine, Nanjing, China; 3 Laboratory of Genetics and Physiology, National Institute of Diabetes, Digestive and Kidney Diseases, National Institutes of Health, Bethesda, MD, United States of America; 4 Regenerative Medicine Research Center, West China Hospital, Sichuan University, Chengdu, Sichuan, China; University of Bern, SWITZERLAND

## Abstract

Acupuncture is reported to be effective in treating obesity related illnesses, but its mechanism is still unclear. To investigate this mechanism we applied electro-acupuncture (EA) in a mouse model of obesity and used RNA-seq to identify molecular consequences. Deletion of the transcription factor STAT5 from neurons (*Stat*5NKO) led to obesity. Acupuncture, in turn, reduced body weight and the ratio of epididymal white adipose tissue (Epi-WAT) to body weight, and it also decreased plasma concentrations of glucose, triglyceride, and cholesterol. In addition, EA increased cold endurance of *Stat*5NKO obese mice. EA reversed altered gene expressions in the hypothalamus and Epi-WAT, especially in the hypothalamus in *Stat*5NKO obese mice. This study provides, for the first time, insight into genomic networks of obesity and their modulation by electro-acupuncture, which in turn reveals potential mechanisms that explain acupuncture-induced weight-loss.

## Introduction

Obesity is characterized by fat accumulation resulting from excess energy intake and insufficient energy expenditure, leading to abnormal excessive storage of triglycerides in adipose tissue. It is considered as one of the highest risk factors for type 2 diabetes, cardiovascular disease, hypertension, cancer, and increased morbidity and mortality rates [1–4]. Obesity epidemic has been on the rise for four decades, and the number of obese individuals is estimated to reach 1.12 billion by 2030 [5,6]. However, most of the current anti-obesity drugs and therapies have failed to achieve adequate weight control in patients, due to side effects such as mood changes, suicidal thoughts, and gastrointestinal and cardiovascular complications[7].

Acupuncture, as an important treatment approach of Traditional Chinese Medicine, has been applied to treat obesity in China for centuries, and it has been approved by the National Institutes of Health and the WHO as a useful procedure for a wide range of conditions[8]. In addition to effective reduction in parameters such as body weight, body mass index, obesity degree or waist/hip ratio, many clinical trials show that acupuncture treatment can improve obesity-related complications, including dyslipidemia, inflammation, and plasma leptin concentration, suggesting that acupuncture is a safe and effective therapy for patients with obesity [8–12]. Experimental research also show a reduction in the body weight of high fat diet (HFD)-induced obese rats with acupuncture treatment on Zusanli (ST36) and Neiting (ST44) acupoints, along with a significant decrease in their plasma leptin levels [13]. Additionally, serum levels of TNF-alpha, IL-6, and IL-1, which contribute to inflammatory response in the HFD-induced obesity model have decreased with acupuncture[14]. EA treatment can also attenuate hepatic lipid accumulation via AMP-activated protein kinase (AMPK) activation in obese rats[15]. Our previous work reveals that acupuncture can increase brown adipose tissue marker expression and cold endurance ability in HFD-induced obese mice[16]. However, further prospective experiments are needed to uncover the underlying peripheral and central mechanisms of acupuncture on obesity due to its complexity.

Genetic research of human obesity show that, in the rare monogenic forms of obesity, all the mutations lie in the central nervous system (CNS), which regulates the body’s energy homeostasis [17,18]. STAT5 is one of the most promiscuous members of signal transducers and activators of transcription (STATs) family; it is highly expressed in distinct populations of hypothalamic neurons[19]. STAT5 has been reported as a vital factor in the adipogenesis and obesity. It is phosphorylated at its tyrosine site during the process of adipogenesis then translocated into the nucleus to activate downstream gene expressions [20,21]. Constitutively active STAT5 can substitute growth hormone to promote adipogenesis of preadipocytes, but its anti-sense oligonucleotides can attenuate this effect [22,23]. Our previous *in vivo* research found that both male and female mutant mice, whose *Stat*5*a/b* loci in the CNS were deleted (*Stat*5NKO), developed severe obesity, accompanied by hyperphagia, hyperleptinemia, impaired thermal response to the cold, and insulin resistance [24]. But how the central STAT5 signaling regulates appetite, thermogenesis, as well as peripheral lipid profile and adipogenesis, remains unclear.

Following the previous research results, we explored the potential role of central *Stat*5 in the acupuncture treatment on obesity in this study. First, we applied acupuncture treatment on *Stat*5NKO obese mice, at ST36 and ST44 acupoints, to evaluate the effectiveness of acupuncture. Second, we employed RNA sequencing (RNA-seq) to generate gene expression profiles in the white fat tissue and the hypothalamus of the *Stat*5NKO obese mice with or w/o acupuncture treatment, to explore the characteristics of gene expression in these mice, and the differentially expressed genes (DEGs) due to acupuncture. Our data suggest that STAT5 in CNS plays different roles in the hypothalamus and white fat tissue during gene transcription activities, and that acupuncture could regulate a large amount of DEGs toward their normal expression levels, especially in the hypothalamus. Thus, the weight loss effect of acupuncture may be attributed to its functional gene regulatory mechanisms.

## Materials and methods

### Animals and grouping

Central specific *Stat*5-deleted mice (*Stat*5NKO mice) were generated as previously described (Yongzhi Cui; 2004). We briefly crossed mice bearing the Cre recombinase expressed under the control of the *Nestin* promoter (*Nestin*-*Cre*) (gift from Dr. Hanover at NIH, USA) with mice harbouring *loxP-*flanked *Stat*5*a/b* alleles (*Stat*5*fl/fl* mice) (provided by Dr. Hennighausen at NIH, USA). *Stat*5fl/fl and *Stat*5NKO mice were housed at 23 ± 1°C in a 12 hr light/dark cycle with free access to water and chow food. Western blot was used to confirm the deletion efficiency of the *Stat*5NKO mice (S1 Fig). 12-week old *Stat*5NKO mice with body weight 20% heavier than that of *Stat*5*fl/fl* mice were selected as obese mice and divided into *Stat*5NKO group, *Stat*5NKO*+*EA group, the same week-old *Stat5fl/fl* mice were divided into *Stat*5*fl/fl* group and *Stat*5*fl/fl+EA* group, each group has 10 mice. Body weight and food consumption in all groups were recorded once a week. This study was approved by the Institutional Animal Care and Use Committee of Nanjing University of Chinese Medicine, and all procedures were conducted in accordance with the guidelines of the National Institutes of Health Animal Care and Use Committee.

### EA intervention

Mice were put under isoflurane anesthesia for three minutes, then received EA treatment and restriction separately. Mice in the *Stat*5NKO*+*EA and *Stat*5*fl/fl+*EA groups received EA treatment with a fixed instrument at Zusanli (ST36) and Neiting (ST44) one side of the body at a time (both sides were used alternatively), once a day, 6 times per week, total 4 weeks. The needles (Beijing Zhongyan Taihe Medical Instrument Co., Lot:191526) with a diameter of 0.18 mm and a length of 10mm, were inserted into these two acupoints, and electrical currents was provided through Han’s Acupoint Nerve Stimulator (Han Acuten, WQ1002F, Beijing, China) with a frequency of 2/15Hz at an intensity level of 0.4–0.6 mA to produce slight twitches in the limbs, for a total stimulation period of 30 minutes. The localization of acupoints followed the standard in Experimental Acupuncturology (Lin and Wang, 1999). ST36 is located at the anterior tibia muscle, about 3 mm below the knee joint. ST44 is located between the second and third phalanges on the toe (S2 Fig). The mice in the *Stat*5NKO group and the *Stat*5*fl/fl* group were restrained for 30 mins without EA stimulation. The EA procedure was carried out with extremely gentle operation to avoid any unnecessary stimulus and stress to the mice. Water, padding and food (monitored weight every time) were changed after restriction and EA treatment every day.

Mice were euthanized with intravenous injections of high-dose pentobarbitone. The hypothalamus and adipose tissue, including epididymis white adipose tissue (Epi-WAT), inguinal white adipose tissue (Ing-WAT), and brown adipose tissue (BAT), located at the scapular area of mice, were dissected, weighed and immediately snap-frozen in liquid N_2_ and stored at -80°C for further analysis.

### Detection of plasma triglyceride, cholesterol, LDL, leptin, and glucose level

Concentrations of circulating triglyceride (TG), total cholesterol (TC), low-density lipoprotein (LDL) (F001-1 for TG, F002-1 for cholesterol, A113-1 for LDL; Jiancheng Institute of Biotechnology), and leptin (CRYSTAL CHEMINC, #90030) were detected by ELISA according to the manufacturer’s recommendations. 40 μl standards or mouse serum were added into a 96 well coated plate with 10 μl of biotin labeled antibody and incubated at 37°C for 30 min. After five washings, HRP Conjugate reagent was mixed in for another 60 min at 37°C. Then, the samples were incubated with Chromogen solutions for 15 min at 37°C. OD values were detected at 450 nm by using Multiskan FC (Thermo scientific, USA) following the addition of the stop solution. TG, TC, LDL, and leptin levels were quantified by a standard curve. The concentration of glucose in plasma was measured using a glucometer (Johnson & Johnson Biological Devices, China) and glucose test strips (One Touch Utra, Lot: 3836186).

### Histology analysis

The tissues were fixed in 4% paraformaldehyde and embedded with paraffin. Haematoxylin and eosin staining (H&E staining) were performed as previously described on 6-μm Epi-WAT sections. Images for analysis were acquired using a light microscope (Nikon, Japan). For adipocyte diameter analysis, five images per animal were collected using Image J software (National Institutes of Health Bethesda, MD, USA). We manually circled adipocytes (more than 250 cells per animal) and measured adipocyte diameters[25].

### Mice cold endurance experiment

For cold room experiments, mice were still singly housed and freely access to water and food (also monitored weight before), we also put thick padding in the cage to keep warm at the same time. Mice were fasted and transferred to a cold room at 4°C for 24 hours. Body temperatures were monitored at different intervals (0, 3h, 6h, 12h, 24h) throughout the day with a rectal probe by via an electro-thermometer (TH212, Hongauchengyun, China).

### RNA-seq and computational analysis for RNA-seq data

To uncover the potential role of central STAT5 in the development of obesity, as well as the underlying molecular mechanism of EA treatment on obesity, RNA-seq for the hypothalamus and Epi-WAT were performed using next generation high-throughput sequencing.

Total RNA of the hypothalamus and Epi-WAT were isolated using Trizol® Reagent (Invitrogen, 15596018). RNA concentration was quantified by Qubit® 2.0 fluorometer (Invitrogen) with Qubit® RNA BR assay kit (Invitrogen, Q10211). RNA quality control (Agilent,1309) was performed using Agilent 2100 Bio-analyzer (Agilent Technologies, Inc. CA, USA) according to the manufacturer’s protocols.

For RNA-seq, RNA library was prepared according to the TruSeq RNA Sample Preparation v2(Illumina; 15025062) protocol, followed with cluster generation and sequencing using cBOT Multiplex re-hybridization plate and TruSeq SBS kit V3(Illumina; 15021668). Sequencing was performed using Illumina Hiseq 2000 (Illumina, USA). Data analysis was performed as previous described. Raw FASTQ files were extracted from Illumina BCL using the Illumina CASAVA program and then aligned to the mouse reference genome (UCSC mm9 assembly) using the TopHat program. The Cufflinks program was used to assemble individual transcripts, and the Cuffdiff program was applied for differential transcript expression analysis. The genes’ functional annotation and pathways were analyzed using the DAVID Bioinformatics Resources [22]. Heat map was generated by the Cluster and TreeView software. Genes with lower than one FPKM (average fragments per kilobase of transcript per million fragments mapped) were filtered out. Up-regulation and down-regulation were defined as a relative transcription level above Log_2_ fold change (FC) ≥ |±1| and *P* value <0.05. The raw RNA-seq data were deposited to NCBI’S Read Archhive (SRA) database and the accession number is GSE100599.

### qRT-PCR

Four micrograms of RNA was converted to cDNA using reverse transcriptase and random primers (#1621, Thermo, Waltham, MA, USA). The primer sequences are shown in S1 Table. For the PCR analysis, the samples were amplified with duplication using SYBR Green (Thermo, #PC4602) with 200 nM of gene-specific primers and run on the CFX amplifier (MX3000P, Stratagene, La Jolla, CA, USA) using the manufacturer’s protocol. Data were analyzed by the threshold cycle (Ct) relative-quantification method.

## Results

### EA treatment restored Stat5NKO-induced obese phenotype

**Fig 1:**
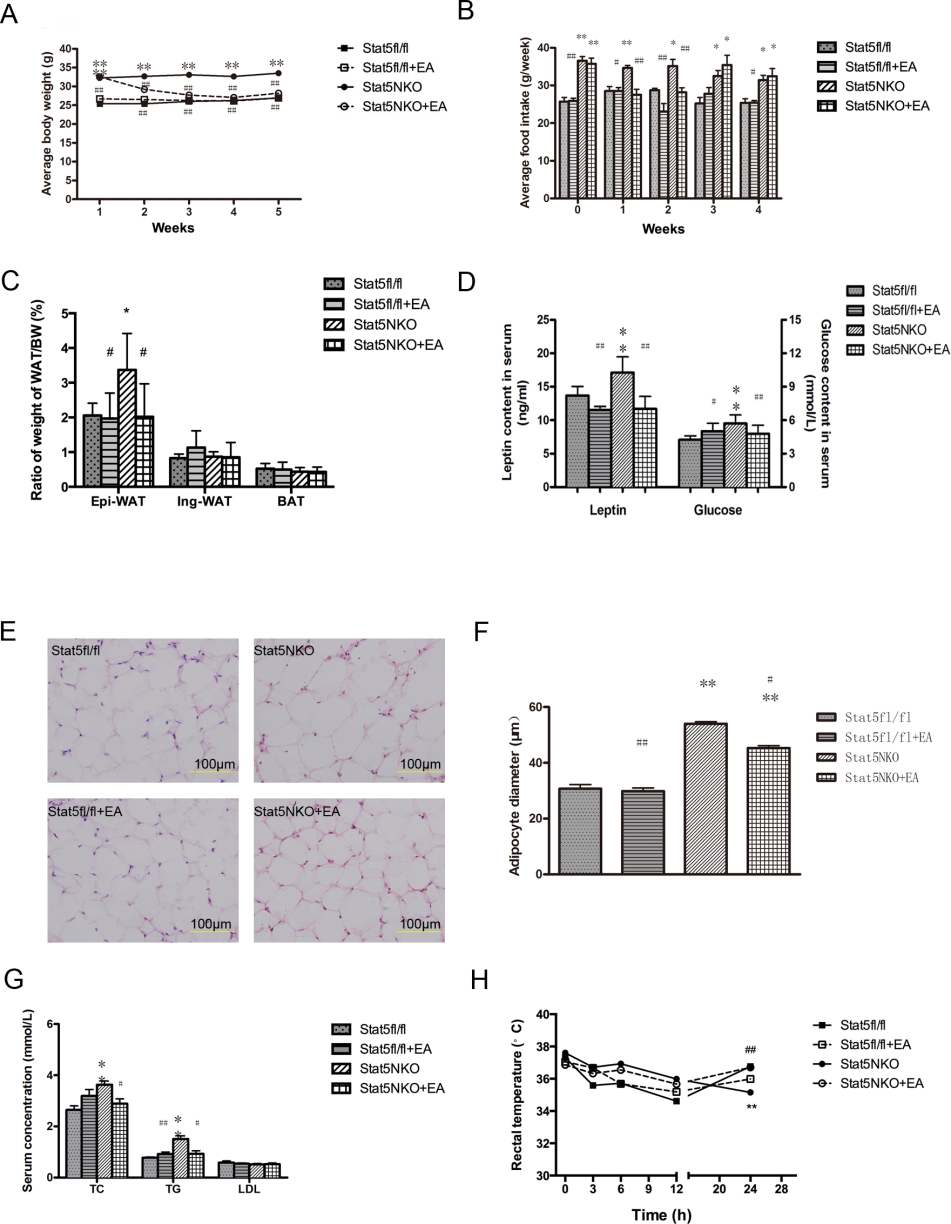
EA treatment restored *Stat*5NKO-induced obese phenotype, improved energy metabolism and cold tolerance in the *Stat*5NKO obese mice. (A) Observation of body weight in the four groups, n = 10 in each group. (B) Measurement of food consumption, n = 10 in each group. (C) Ratio of epididymal and inguinal white adipose tissue, brown adipose tissue weight to mouse body weight, n = 10 in each group. (D) Mouse serum leptin was detected by ELISA, glucose level was measured by using glucometer after 4-weeks electro-acupuncture treatment, n = 10 in each group. (E &F) EA treatment decreased *Stat*5NKO obese mice adipocyte size. H&E staining of Epi-WAT (E) together with average adipocyte diameter (F) in EA and control group mice, n = 5 in each group. (G) EA treatment decreased *Stat*5NKO obese mice’s serum TG and TC levels. Serum cholesterol (TC), triglyceride (TG) and low density lipoprotein (LDL) level were measured by ELISA. n = 10 in each group. (H) EA treatment increased the ability of cold endurance in the *Stat*5NKO obese mice. Mice were settled in 4°C cold room, rectal temperature were measured after 3, 6, 12 and 24 hours; n = 6 in each group. *P < 0.05, ** P<0.01 vs the *Stat*5fl/fl group; ^#^ *P*<0.05, ^##^ *P*<0.01 vs the *Stat*5NKO group. All data were expressed as means ± SD, and each experiment was repeated 3 times independently.

During the four weeks of EA intervention, body weight and food consumption of the mice were recorded weekly. The results showed that EA significantly reduced the *Stat*5NKO mice’s body weight throughout the duration of treatment (*P*<0.01)–even as low as that of the *Stat*5 *fl/fl* group after two weeks of treatment. On the other hand, EA treatment did not change the body weight of the *Stat*5*fl/fl* mice (Fig 1A). We observed increased food consumption in the *Stat*5NKO mice compared with the *Stat5 fl/fl* group, and a subsequent decrease in food consumption following EA treatment in the *Stat*5 NKO mice during the first two weeks of treatment (*P*<0.05) that increased again during the last two weeks (Fig 1B). Additionally, we harvested the epididymis white adipose tissue (Epi-WAT), inguinal white adipose tissue (Ing-WAT), and brown adipose tissue (BAT) from each group and measured their weight. The ratio of each type of adipose tissue to the body weight was calculated. The results indicated that the Epi-WAT volume in the *Stat*5NKO group was larger than that in the Stat5*fl/fl* group, and EA significantly decreased this ratio (*P*<0.05) (Fig 1C and S3 Fig). Serum leptin and glucose levels were increased after the deletion of neural *Stat*5 gene (*P*<0.01) but were restored to baseline levels similar to those in the *Stat*5*fl/fl* mice following EA treatment (Fig 1D). Moreover, H&E staining confirmed that lipid droplets in the *Stat5*NKO mice were bigger than those in the *Stat*5*fl/fl* mice (*P*<0.01), and EA treatment notably decreased size of adipocytes in the *Stat*5NKO mice (*P*<0.01) but did not affect the *Stat*5*fl/fl* mice (Fig 1E and 1F).

### EA treatment improved energy metabolism and cold tolerance in the *Stat*5NKO obese mice

The plasma concentrations of triglyceride (TG), cholesterol (TC), and Low density lipoprotein (LDL) were detected using ELISA. In comparison to the *Stat*5*fl/fl* group, TC and TG levels in the *Stat*5NKO group were both significantly elevated (*P*<0.01) initially but were completely reversed to normal levels after four weeks of EA treatment. We did not observe a difference in LDL concentrations among these four groups (Fig 1G).

To investigate whether the improved lipid metabolism by EA is related to WAT browning or elevated thermogenesis ability, Six mice from each group were submitted to a cold endurance experiment. We recorded rectal temperatures of the mice at 0, 3, 6, 9, and 24 hours after placing them into a cold room. The results showed that *Stat*5NKO mice’s rectal temperatures consistently decreased during the observation period, whereas the decreased temperatures of *Stat*5*fl/fl* mice were elevated after 12 hours in the cold room and reverted to the normal level. We also observed that EA treatment increased rectal temperature of the *Stat*5NKO mice at the 24-hour mark but did not affect the *Stat*5*fl/fl* mice (Fig 1H), indicating that EA treatment could increase the *Stat*5NKO obese mice’s ability to adapt to cold temperatures.

### EA treatment changed genome-wide expression profiles of hypothalamus and Epi-WAT in the *Stat*5NKO obese mice

#### Genome-wide analysis of *Stat*5NKO mice

To uncover the potential role of central STAT5 in the development of obesity, as well as the underlying molecular mechanism of EA treatment on obesity, RNA-seq for the hypothalamus and Epi-WAT were performed using next generation high-throughput sequencing. Compared with the *Stat*5*fl/fl* group, 420 genes were differentially expressed in the hypothalamus upon the loss of *Stat*5 in the central neuron system, in which 80% (336) of the genes were up-regulated and 20% (84) of the genes were down-regulated (Table 1).

**Table 1:** Differentially expressed genes(DEGs) with a log2(FC)>|±1| and p value<0.05.

DEGs	Hypothalamus		Epi-WAT	
	NKO vs fl/fl	EA vs NKO	NKO vs fl/fl	EA vsNKO
Up-regulated	336 (80%)	35 (8.6%)	1047 (48.6%)	361(79.5%)
Down-regulated	84 (20%)	370 (91.4%)	1106 (51.4%)	93 (20.5%)
Total	420 (100%)	405 (100%)	2153(100%)	454 (100%)

NKO: *Stat*5NKO group; fl/fl: *Stat*5fl/fl group, EA; *Stat*5NKO+EA group.

**Fig 2:**
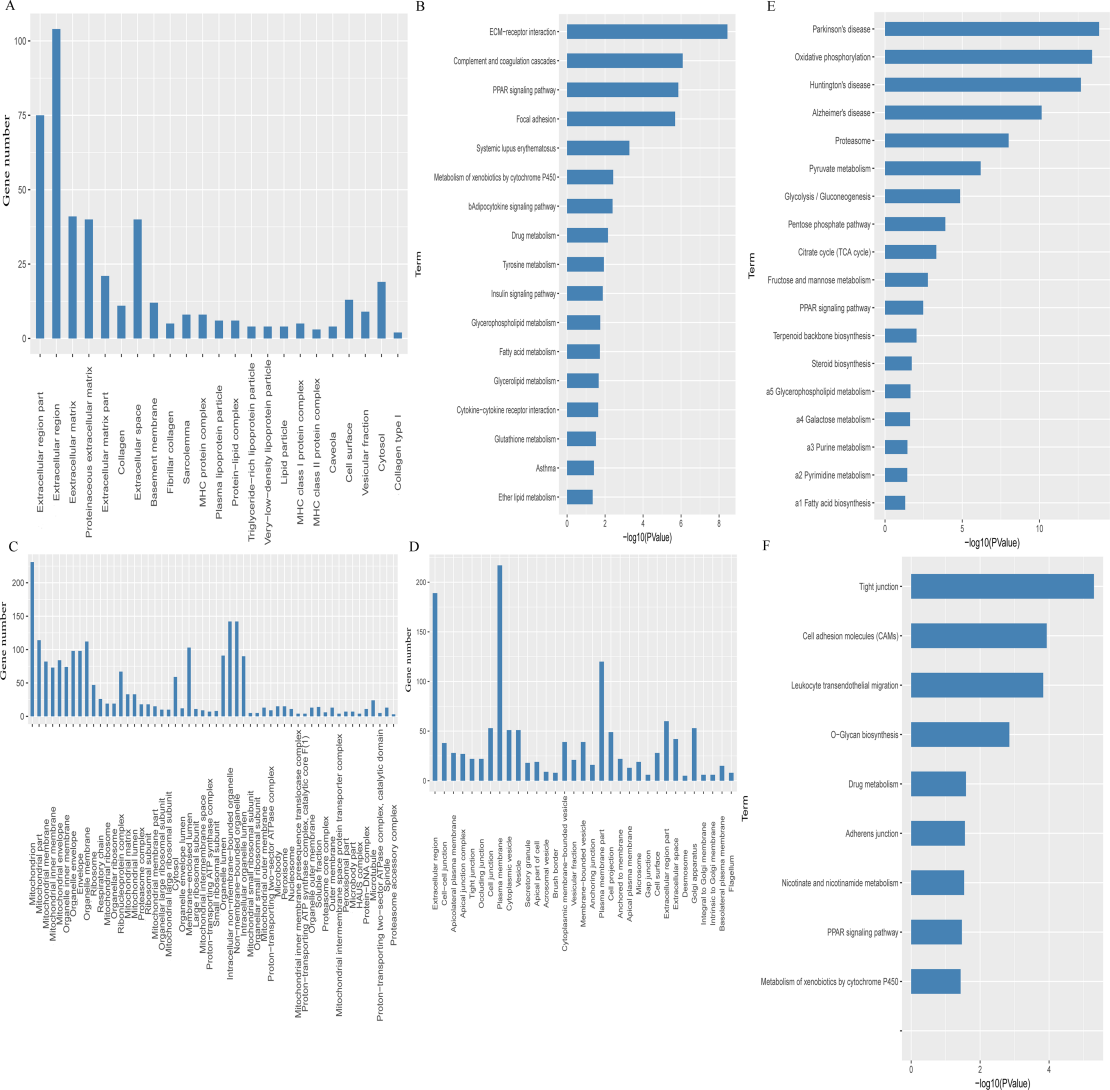
GO annotation and KEGG pathways analysis of up- or down-regulated genes in the *Stat*5NKO mice hypothalamus and Epi-WAT. (A–F). (A) Cellular components annotation results of the 388 up-regulated genes in *Stat*5NKO mice’s hypothalamus shown in Table 1. (B) Major KEGG pathways of the 338 up-regulated genes in *Stat*5NKO mice hypothalamus shown in Table 1. (C) Cellular components annotation results of the 1047 up-regulated genes in *Stat*5NKO mice Epi-WAT draw from Table 1. (D) Cellular components annotation results of the 1106 down-regulated genes in *Stat*5NKO mice Epi-WAT draw from Table 1. (E) KEGG pathways result was drawn from the 1047 genes shown in Table 1; (F) KEGG pathways result was drawn from the 1106 genes shown in Table 1.

Gene ontology (GO) annotation indicated that the up-regulated genes in the hypothalamus of the *Stat*5NKO mice were mainly expressed in the extracellular region, basement membrane, plasma lipoprotein particle, and cytosol (Fig 2A); and these genes were significantly enriched in 140 biological processes, which were functionally clustered to extracellular matrix organization, response to hormone and insulin stimulus, lipid and saccharide metabolic process, fat cell differentiation, and positive regulation of adaptive and innate immune responses. Kyoto Encyclopedia of Genes and Genomes (KEGG) pathway analysis confirmed that these up-regulated Differentially Expressed Genes (DEGs) belonged to extracellular matrix (ECM)-receptor interaction, complement and coagulation cascades, PPAR (Peroxisome Proliferator Activated Receptor) signaling pathway, adipocytokine signaling pathway, insulin signaling pathway, and several substance metabolism, which are closely related to lipids metabolism (Fig 2B). On the other hand, the down-regulated genes in the hypothalamus were also mainly located in extracellular region but were involved in the regulation of defense response to bacterium and blood circulation, and no significantly enriched pathway was detected.

From the above analysis, we observed that a large number of genes and functional pathways involved in lipid metabolism, adipogenesis, insulin signaling, and immune system were dependent on the STAT5 molecule in the CNS. To investigate the extent to which central STAT5 can affect peripheral energy metabolism and adipogenesis, we further measured gene profiling in the Epi-WAT. In the Epi-WAT, 1047 (48.6%) genes were up-regulated and other 1106 (51.4%) genes were down-regulated (Table 1). In contrast to the hypothalamus, the up-regulated genes in the Epi-WAT were enriched in intracellular parts, such as mitochondrion, ribosome, respiratory chain, ribonucleoprotein complex, proton-transporting ATP synthase complex, and proteasome complex (Fig 2C), which are mainly involved in directly regulating cell metabolism processes, including the carbohydrate catabolic process and fatty acid, cholesterol, sterol, triglyceride, neutral lipid, acylglycerol, and ether biosynthesis. Additionally, these genes are also involved in the energy, cofactor, and nucleotide metabolism processes, mediating oxidative phosphorylation, chromatin assembly, DNA packaging, and RNA processing. KEGG pathway analysis suggested that these up-regulated genes were enriched in obesity-related pathways, including glycolysis, TCA (Tricarboxylic acid) cycle, fructose and mannose metabolism, PPAR signaling pathway, steroid biosynthesis, and fatty acid biosynthesis (Fig 2E). On the other hand, the down-regulated DEGs were located in the extracellular region, cell junction, plasma membrane, cytoplasmic vesicle, and Golgi apparatus (Fig 2D). Besides lipid metabolism and transport-related biological processes, GO annotation showed that these genes were mainly functionally clustered to spermatogenesis and fertilization, defense response to bacteria, cell adhesion, cell motion, ion and carboxylic acid transport, and chordate embryonic development. KEGG pathway analysis indicated that these DEGs significantly regulated cellular community signals (e.g. tight junction, CAMs, and adheren junctions), immune response (e.g. leukocyte transendothelial migration), PPAR signaling pathway, O-glycan biosynthesis, and drug metabolism (Fig 2F).

**Fig 3:**
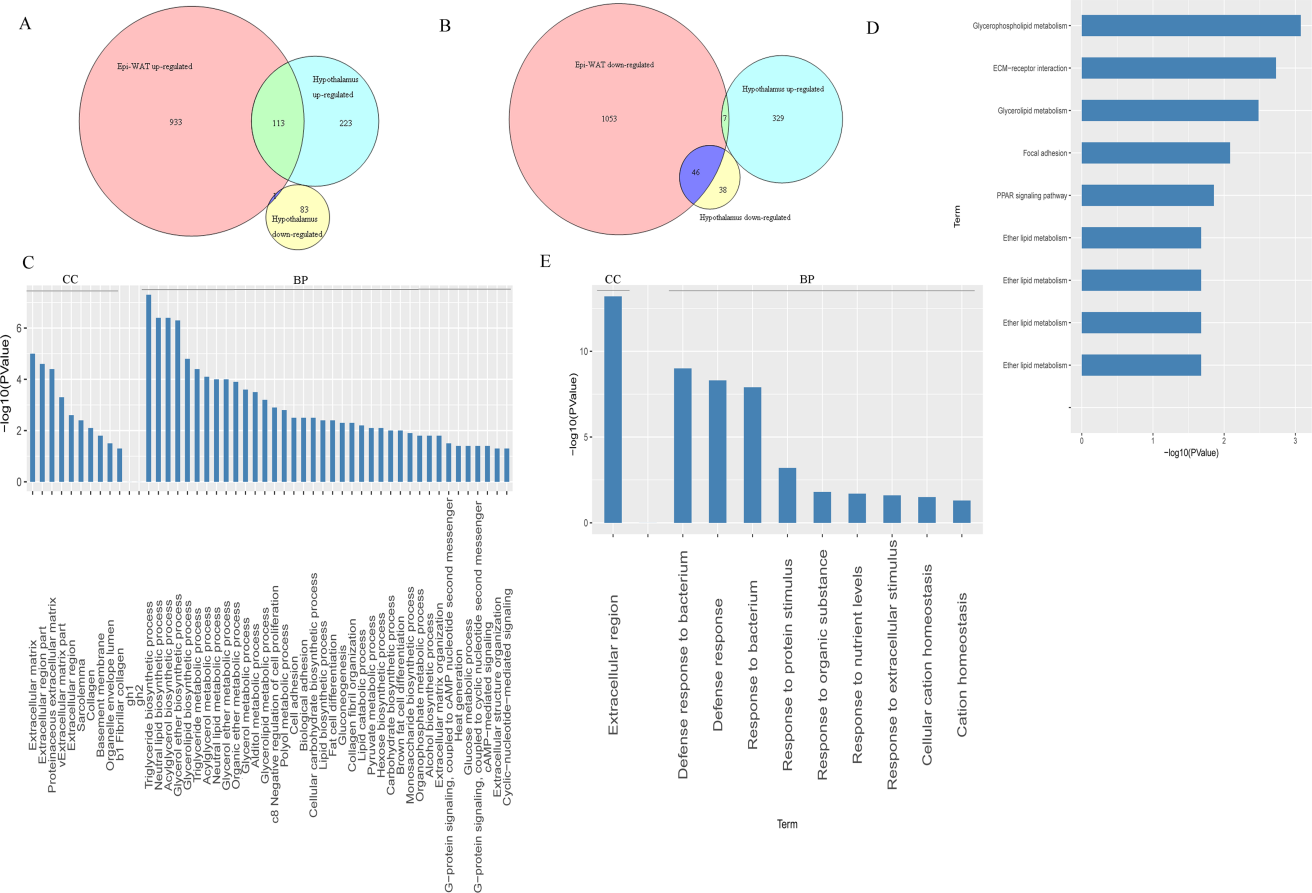
Venn diagrams and GO annotation analysis of co-regulated genes in both central (hypothalamus) and peripheral (Epi-WAT) tissues (A-D). (A) Venn diagrams were drawn based on the RNA-seq data sets. Red circles indicate the numbers of up- or down-regulated genes in Epi-WAT (*vs*. *Stat*5fl/fl group); green circles represent the numbers of up-regulated genes in the hypothalamus (*vs*. *Stat*5NKO group). Blue circles represent the numbers of down-regulated genes in the hypothalamus (*vs*. *Stat*5NKO group). (B) GO annotation of co-upregulated genes in the *Stat*5NKO obese mice’s hypothalamus and WAT. (C) KEGG pathway for the co-upregulated genes in the hypothalamus and WAT of the *Stat*5NKO obese mice. (D) GO annotation results of co-down regulated genes in the hypothalamus and WAT of the *Stat*5NKO obese mice.

We further analyzed the lists of up- and down-regulated DEGs of the hypothalamus and Epi-WAT using Venn diagrams. The results showed that 114 genes were co-up-regulated both in the hypothalamus and Epi-WAT. Only one gene, Ret, a member of the cadherin superfamily, which encodes one of the receptor tyrosine kinases and transduces signals for cell growth and differentiation, was up-regulated in WAT but down-regulated in the hypothalamus (Fig 3A and 3B). These 114 co-up-regulated genes were located in the extracellular matrix, collagen, and organelle envelope lumen, and involved in the lipid biosynthetic process (e.g. triglyceride, neutral lipid, acylglycerol, glycerol ether, and glycerolipid), cellular carbohydrate biosynthetic processes (e.g. gluconeogenesis, hexose biosynthetic, and pyruvate metabolic process), and extracellular matrix organization (Fig 3C). KEGG analysis showed that they mainly participated in glycerophospholiplid metabolism, glycerolipid metabolism, PPAR signaling pathway, and ether lipid metabolism, which contributed to lipid metabolism (Fig 3D). To provide more detailed information, we listed the top 50 DGEs with the highest fold changes in hypothalamus and Epi-WAT in S2–S5 Tables. We found that 29 out of the top 50 up-regulated DEGs in the hypothalamus also were up-regulated in Epi-WAT, in which 8 genes (1700047G03Rik, Mogat2, Igfals, Agpat2, Gys2, Odf3l1, Gm6484, Ffar2) were also listed in the top 50 up-regulated genes in Epi-WAT. Meanwhile, our analysis of the down-regulated genes found that 46 genes were co-down-regulated in hypothalamus and Epi-WAT. Although these genes were not significantly enriched by the KEGG pathway terms, GO analysis revealed that they participated in defense responses to bacteria, protein stimuli, organic substances, and nutrient levels. 25 and 14 genes out of the top 50 down-regulated genes in the hypothalamus and Epi-WAT, respectively, were included in the co-down-regulated gene list, in which 10 genes were common genes (down-regulated in both hypothalamus and Epi-WAT), including Adam7, 9230104L09Rik, Teddm1, 2210415F13Rik, Defb25, Defb30, Spink2, Wfdc10, 5830403L16Rik, and Ly6g5b. In addition, other eight genes, Mmp7, Hspb7, Hba-a1/a2, Crip1, Ccl6, Krt19, Ccl9, and F13a1 were up-regulated in hypothalamus but down-regulated in the Epi-WAT (Fig 3E).

#### EA treatment reversed the abnormal gene expressions in the *Stat*5 NKO obese mice

**Fig 4:**
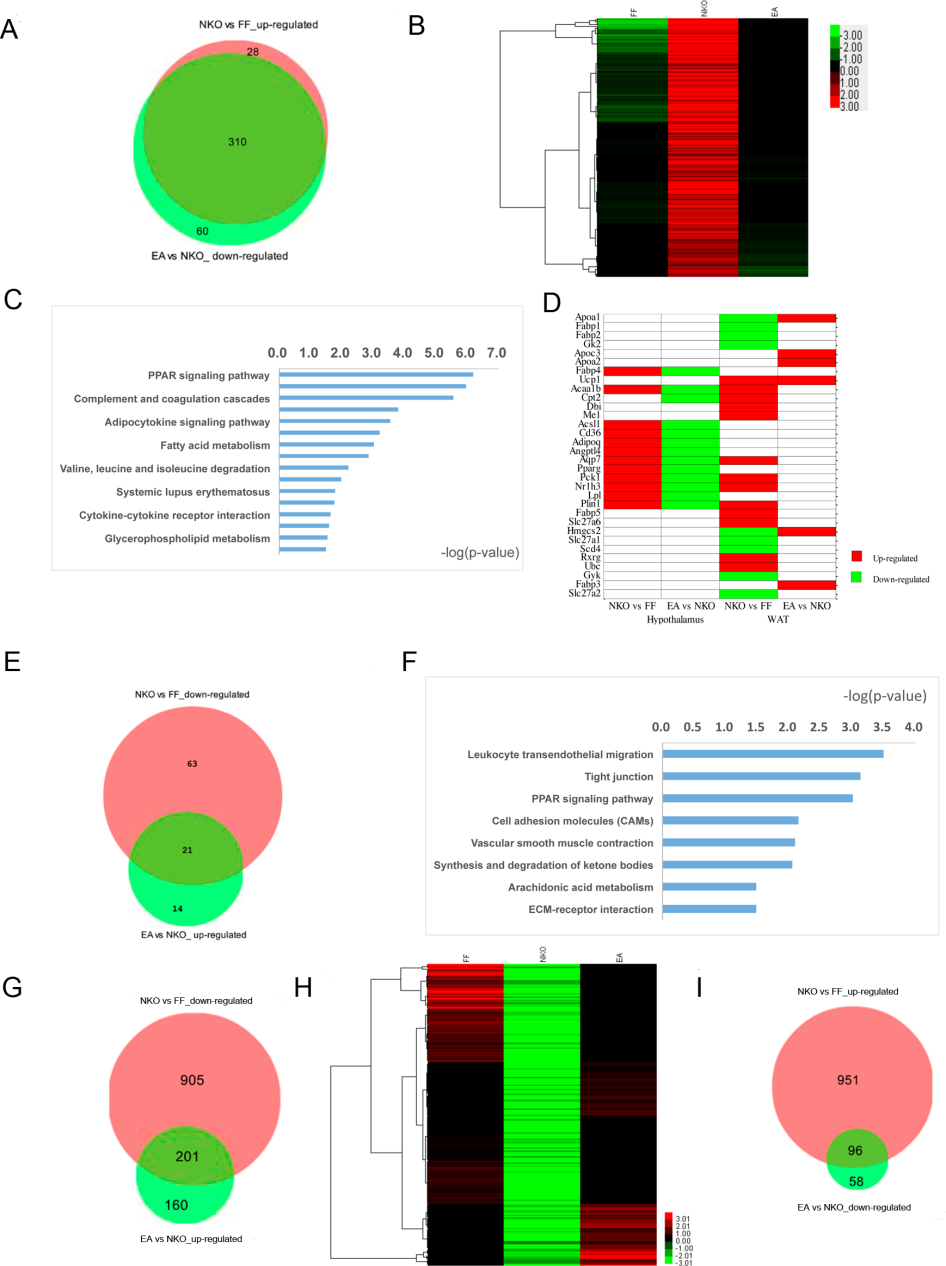
EA treatment altered the abnormally expressed genes both in the hypothalamus (A-E) and Epi-WAT of *Stat*5NKO mice (F-I). (A) Overlapping area represents upregulated gene number (310) in the *Stat*5NKO group (*vs*. the *Stat*5fl/fl group) but decreased in the EA group; (B) The heatmap was created using the FPKM values of the 310 overlapping genes in 5A; (C) Pathway analysis of the 310 overlapping genes in 5A. (D) The expression levels of known genes involved in PPAR signaling pathway were changed in the *Stat*5NKO group (*vs*. the *Stat*5fl/fl group) and some were reversed by EA; (E) Overlapping area represents down-regulated gene number (21) in the *Stat*5NKO group(*vs*. the *Stat*5fl/fl group) but increased in the EA group. (F) Pathway analysis for the 201 co-regulated genes in Epi-WAT. (G) &(I) Venn diagrams were drawn based on the RNA-seq data sets. Red circles indicate the numbers of down- or up-regulated genes in the *Stat*5NKO group (*vs*. *Stat*5 fl/fl group); green circles in (G, I) represent the numbers of up- or down-regulated genes in the EA group (*vs*. *Stat*5NKO group). (H) The heatmaps were created with down-regulated genes in the *Stat*5NKO group and up-regulated genes in the *Stat*5NKO+EA group. Expression levels of up-regulated and down-regulated genes are represented in red and green colors, respectively.

To explore the potential mechanism of EA treatment on weight control, we compared the gene expression profiles of *Stat*5NKO obese mice with that of the EA treated or untreated mice. In the hypothalamus, 370 genes were down-regulated and 35 genes were up-regulated with EA treatment (Table 1). Surprisingly, in the down-regulated genes, 310 also showed in the up-regulated gene list (338) of the *Stat*5NKO group, and the expression levels of most of these 310 genes were decreased after EA treatment, to baseline levels (similar to those of the *Stat*5*fl/fl* group) (Fig 4A and 4B). Moreover, based on the top 50 DEG list, 41 common genes whose expression levels were up-regulated in the *Stat5*NKO group were dramatically decreased after EA treatment, and most of these genes were closely related to the development of obesity, such as Gh, Prl, Lep, Igfals, Plin1, Agpat2, Mogat2, and Cidec (S6 Table). KEGG pathway analysis showed that EA down-regulated 370 genes were enriched in 16 pathways, including substance metabolism–especially lipid metabolism–such as PPAR signaling, adipocytokine signaling, fatty acid metabolism, insulin signaling, and glycerophopholipid metabolism. Moreover, immune function related pathways were also regulated by EA, such as complement and coagulation cascades, cytokine-cytokine receptor interaction, and systemic lupus erythematosus. Compared with the up-regulated gene pathways in the *Stat*5NKO group, except for the valine, leucine, and isoleucine degradation pathways, EA treatment modified 15 out of 16 pathways, and PPAR signaling pathway were listed as the most enriched pathway in the *Stat*5NKO*+*EA group (Fig 4C). Examining the PPAR signaling pathway revealed that most of the involved DEGs were directly reversed by EA (Fig 4D). On the other hand, 21 out of the 35 up-regulated genes in the *Stat*5NKO*+*EA group were those listed in the down-regulated genes (84) from the *Stat*5NKO group. Among these genes (Fig 4E), FPKM values of 7 genes, which are associated with immune system and the response to hormone (AY761185, Cst11, Defb20, Defb28, Defb48, Lcn8, Lcn9) were decreased to zero by the deletion of central *Stat5*, whereas EA treatment reversed their expression levels, and the other 13 genes, such as Gpx5, 9230104L09Rik, Cst12, and Defb25, appeared in the top 40 down-regulated gene list of the *Stat*5NKO mice (S7 Table). Altogether, our results suggest that EA treatment can specifically reverse differentially expressed genes via the loss of STAT5 in the central nervous system.

In the Epi-WAT, compared to the *Stat*5NKO group, 454 DEGs were affected in the *Stat*5NKO*+*EA group, in which 361 genes were up-regulated, and 93 genes were down-regulated (Table 1). The up-regulated 361 genes were enriched in 8 KEGG pathways, and 4 of these pathways (leukocyte transendothelial migration, tight junction, PPAR signaling pathway, and CAMs) were seen in the *Stat*5NKO induced down-regulated pathways (Fig 4F). Further analysis showed that 201 out of the 361 EA up-regulated genes were on the down-regulated gene list (1106) of the *Stat*5NKO group. Cluster 3.0 analysis indicated that the FPKM values of these genes in the *Stat*5NKO*+*EA group were similar to that in the *Stat*5*fl/fl* group (Fig 4G and 4H). Additionally, 4 up-regulated genes (Ucp1, Cox7a1, Trfr2 and Ptgr1) as a result of loss of *Stat*5 in the CNS were also up-regulated by EA treatment, especially the gene encoding UCP1 (uncoupling protein1), which is involved in energy metabolism. Its FPKM value was nearly zero in the Epi-WAT of the *Stat*5 *fl/fl* mice but was increased to 1.51 in the *Stat*5NKO obese mice, then to 257.39 following EA treatment, becoming the second most EA up-regulated gene (S8 Table). The EA down-regulated 154 genes were only significantly enriched in complement and coagulation cascades, and over half of these genes (96) were in the up-regulated gene (1047) list of the *Stat*5NKO group (Fig 4I). 42 out of these 96 genes were listed as some of the top 50 genes down-regulated by EA in the hypothalamus, and 19 out of these 42 genes were listed as some of the top 50 up-regulated genes in Epi-WAT, such as Gbp1, Fam5b, Dnmt3l, Fmr1, Mogat2, and Tuba1a (S9 Table). Although *Lep* (the gene encoding Leptin) was not included in the top 50 genes down-regulated by EA in the hypothalamus, its FPKM value of the *Stat*5 *fl/fl* mice was 4.33, but was decreased from 58.59 to 4.66 in the *Stat*5NKO+EA group. Our data thus suggest that EA can reverse the STAT5-dependent genes to their baseline levels to a large extent.

### Validation of RNA-seq data

**Fig 5:**
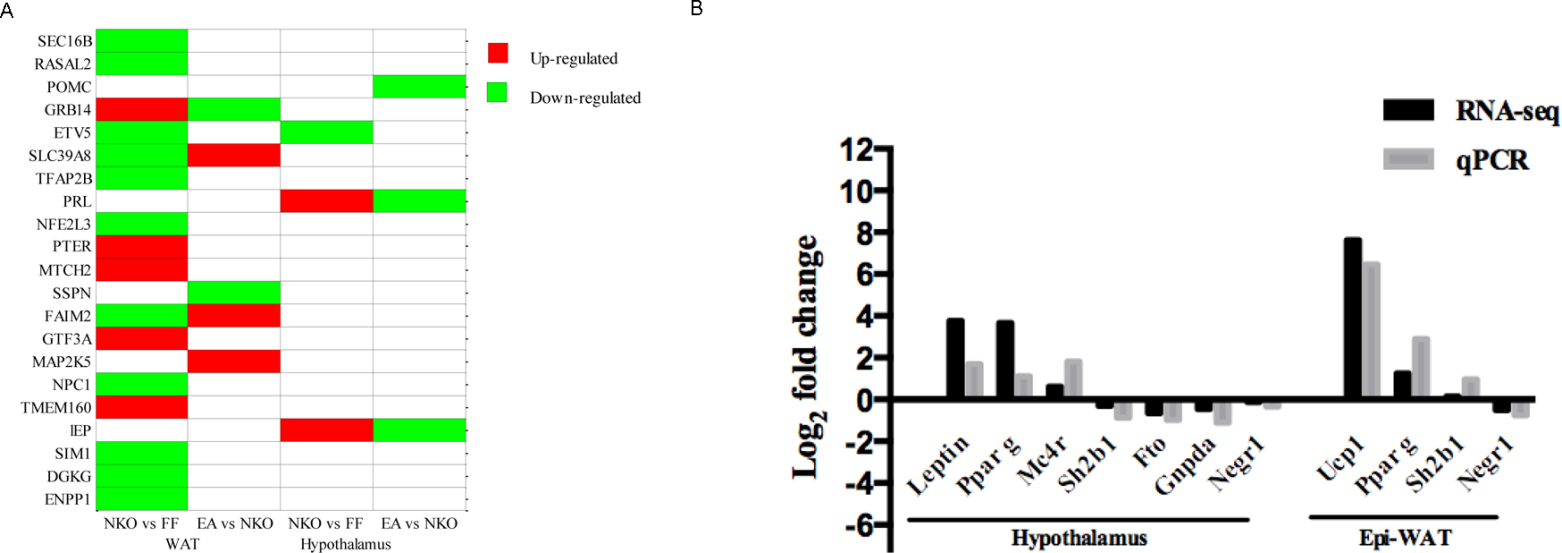
Conformation of RNA-seq data. (A) Comparison of our RNA-seq data and the known genes associated with obesity phenotypes. DEGs in the RNA-seq data with a log2(FC)>|±1| and p value <0.05 were qualified to be analyzed, red color presents up-regulation, green color presents down-regulation. (B) qRT-PCR validation of RNA-seq results. Comparison of fold change (log2) in differential expression values determined by RNA-seq (black) and qPCR (grey) for DEGs, n = 6 for qPCR verification.

To verify our RNA-Seq data, we specifically checked for well-known monogenic obesity phenotype related genes. We found that 21 known genes [18,26,27]appeared on the list for the hypothalamus of *Stat*5NKO mice (Fig 5A), and two of them (Prl, Lep) were up-regulated in the hypothalamus of the *Stat*5NKO group and were decreased by EA, but only one gene (Etv5) was down-regulated due to the loss of *Stat*5. In Epi-WAT, 11 genes (Sec16b, Rasal2, Etv5, Slc39a8, Tfap2b, Nfe2l3, Faim2, Npc1, Sim1, Dgkg, and Enpp1) were down-regulated by the loss of *Stat*5, and two of them (Slc39a8 and Faim2) were significantly up-regulated by EA. Meanwhile, five genes (Grb14, Pter, Mtch2, Gtf3a, Tmem160) were up-regulated in the *Stat*5NKO group, and Grb14 was down-regulated by EA. Furthermore, we detected relative mRNA expressions of several genes by qRT-PCR in a randomly selected subset of samples to further test the validation of these data. The results demonstrated that the tendencies of RNA-seq data and qRT-PCR data were similar (Fig 5B).

## Discussion

Acupuncture has been reported as an effective treatment for a number of obesity-related symptoms, but most of the mechanistic studies focus on the peripheral targets, such as adipose tissue and serum metabolic parameters. However, obesity is a complex metabolism disease in which multiple organs, like the hypothalamus, liver, skeleton muscles, adipose tissue, and pancreas are participating, and multiple problems in lipid metabolism, insulin sensitivity, energy metabolism homeostasis, and chronic inflammation may occur. Increasing evidence indicates that the central nervous system plays an important role in the development of obesity. Our previous study showed that mice with central deletion of *Stat*5 locus developed severe obesity[24] and that electro-acupuncture was effective on HFD-induced obese mice[16]. We then applied acupuncture on the *Stat*5NKO mice in this study to explore the central mechanism of acupuncture on obesity.

We investigated the effectiveness of acupuncture on weight control in the *Stat*5NKO mice. More and more evidence has shown that *Stat*5 plays an important role in the lipid metabolism[20–23]. For example, loss of STAT5 signal in liver leads to hepatosteatosis and impaired liver regeneration, likely through abnormal lipid metabolism[28]. It is also reported that loss of cytokine-STAT5 signaling in the CNS and pituitary gland alters energy balance and leads to obesity, companied with elevated triglyceride and free fatty acids in serum[24]. Recently Baik M. et al described that liver-specific deletion of *Stat*5 gene aggravated fatty liver in response to a high-fat diet in mice[29]. Combined our present study, *Stat*5 is proved to be a key regulator of lipid metabolism, and loss of *Stat*5 in CNS induced weight gain in the mice through various signaling pathways.

For acupoints selection, ST36 and ST44 are classical acupoints for obesity treatment, not only in clinical acupuncture practice, but also in experimental studies. They have been used as a pair to treat obese patients clinically and in animal experiments, including our previous study[16]. For example: Gong, M. et al. found that EA can improve the sensitivity of leptin[13] and can attenuate hepatic lipid accumulation via enhancing AMPK pathway[15], thus contribute to the reduction in body weight in obese rats; Choowanthanapakorn M. et al. thought the weight-loss effect of acupuncture may target at TRPV1 for controlling body weight[30]; Wang F, et al. discovered that the satiety effect of electroacupuncture need arcuate nucleus participate in the hypothalamus of obese rats[31]. Therefore, we selected ST36 and ST44 to be stimulated by electro-acupuncture in the present study.

Our results demonstrated that the *Stat*5NKO mice developed obesity as we reported previously [24], characterized by increased body weight, hyperphagia, hyperleptinemia, hyperglycemia, hypercholesterolemia, and impaired thermal regulation to cold. Electro-acupuncture applied on ST36 and ST44 acupoints decreased the body weight and Epi-WAT/body weight ratio of these *Stat*5NKO obese mice; the adipocyte size, plasma concentrations of leptin, glucose, TC and TG were also decreased after EA treatment. Additionally, the obese mice with EA treatment had significantly improved thermogenic ability, which is associated with activated brown adipose tissue by sympathetic nerve. It is known that ST36 or/and ST44 stimulation excite(s) parasympathetic nerve, however, these conclusions are mainly drew from anesthetized animals, and they were focused on intestinal motor function and electrophysiology. In this study, animals were fixed and accepted acupuncture treatment in conscious, and the rectal temperatures were measured as an index of their thermogenesis ability in cold stimulation. As we know that body temperature is regulated by thermoregulation center, which is located in hypothalamus, and endocrine and sympathetic-adrenal endocrine system were involved in this regulation. Under cold condition, heat is generated by increased CRH, NE, PGE, thyroid hormone, and cAMP, meanwhile energy substance catabolism processes are accelerated. Moreover, Yu’s group found that electroacupuncture at ST36 and SP6 can mitigate the adrenal cortical inhibition induced by etomidate[32]; and Lin reported that EA at ST36 could restore the adrenocortical hypofunction resulting from ICS in dogs[33]. So we believe that the transform of WAT to BAT should be involved in the elevation of heat generation through multiple factors. But further research is needed to reveal the detailed mechanisms of EA on temperature regulation. However, EA applied in the *Stat*5*fl/fl* control mice, showed no change on these observed indexes. All these results suggest that EA can control the body weight of *Stat*5NKO obese mice and help recover obesity-related abnormal lipid and glucose metabolism, as well as energy expenditure–findings consistent with our previous research[16].

Hypothalamus is regarded as an important central regulatory organ for food intake and energy homeostasis through leptin signaling via the transduction of JAK-STAT pathway [34–36]. However, latest research have found that the central activation of PPARγ or arachidonic acid contributes to obesity by direct regulation of indigestive behaviors or impairment of hypothalamic leptin signaling and hepatic energy homeostasis [37]. It seems that the significance of hypothalamus in obesity is far more than what we know. According to our RNA-seq results, over 80% DEGs in the hypothalamus were up-regulated due to the loss of central STAT5 signals, indicating that *Stat*5 might play a role on repressing but not activating gene expressions in the hypothalamus. EA treatment could reverse the abnormal gene expressions both in the hypothalamus and Epi-WAT, especially in the hypothalamus; surprisingly, 91.7% of the up-regulated genes in the hypothalamus in the *Stat*5NKO mice were decreased to normal levels following EA treatment. Functional analysis showed that central STAT5 signal-dependent obesity was ascribed to abnormal expressions of multiple genes involved in lipid metabolism, glucose metabolism, energy homeostasis, and immunity response. Alteration of obesity related signals in the hypothalamus may possibly induce extensive effects on related biological processes in Epi-WAT tissue, and EA treatment could correct the imbalance in energy intake and expenditure in the *Stat*5NKO obese mice. PPAR signaling pathway, which contributes to not only lipid metabolism but also to gluconeogenesis and thermogenesis, was the representative signaling. Our present study showed that this pathway was co-regulated by EA both in the hypothalamus and Epi-WAT. In the hypothalamus, only 12 DEGs were included in this pathway; these genes were up-regulated by the deletion of central *Stat*5 and were involved in adipocyte differentiation (CD36, PPARγ, Angptl4, Fabp4, Adipoq, Plin1), gluconeogenesis (Pck1, Aqp7), fatty acid transport (Lpl, Acsl1, Cd36), cholesterol metabolism (Nr1h3), and fatty acid oxidation (Acaa1b) (Fig 4D). On the other hand, in Epi-WAT, more *Stat*5-dependent DEGs genes (22) were involved in the PPAR signaling pathway. With up-regulation of the genes that encoded the key mediators of lipid metabolism (Rxrg, Slc27a6, and Fabp5), unsaturated fatty acid ubiquitination (Ubc), and lipogenesis (Mei) were also activated in Epi-WAT, besides adipocyte differentiation (Plin1), gluconeogenesis (Pck1, Aqp7), fatty acid transport (Dbi), cholesterol metabolism (Nr1h3), fatty acid oxidation (Acaa1b, Cpt2) in hypothalamus, (Fig 4D). Meanwhile, the down-regulation of Slc27a1, Slc27a2, Fabp2, and Fabp1 might restrict liver and skeletal muscle ketogenesis (Hmgcs2), lipid transport (Apoa1), adipose glycerol kinase activity (Gyk, Gk2), and Scd4-dependent monounsaturated fatty acid synthesis (Fig 4D). Interestingly, our data showed that EA exhibited different functions in gene regulation in the central and peripheral tissues. In the hypothalamus, EA abolished all *Stat*5NKO induced abnormal pro-lipogenesis signals; while in Epi-WAT, EA only reversed a small amount of *Stat*5-dependent DEGs. However, the increased expression of SLC27A2 and FABP3, activated ketogenesis (Hmgcs2), lipid transport (Apoa1, Apoa2, Aopoc3), and thermogenesis (Ucp1) (Fig 4D), accompanied with up-regulation of Cidea, COX7a1, and COX8b, the markers of white adipocyte browning, indicates that EA can increase energy expenditure by promoting the formation of beige cells. This finding is consistent with our previous study on HFD-induced obese mice[16]. But why does EA regulate functional genes much more properly in the CNS than in the peripheral fat tissue? Our current study does not explicitly explain this phenomenon, but we suspect that the complexity in regulating gene expressions in Epi-WAT might be due to some indirect or secondary effects of *Stat5* knockout in the CNS.

Besides the imbalance of substance metabolism, immune system was also reported to be closely associated with the development of obesity. A few studies have suggested that immune cells regulate metabolic homeostasis and are dysregulated in obesity, and that obesity-induced multi-organ chronic low-grade inflammation is recognized as a major cause of insulin resistance[38,39]. In this study, the immune pathway activities were widely detected. For example, complement and coagulation cascades (e.g., C1ra, C7, Thbd, Serpina1b, C3, C4b, Serpina1d, Serping1, Serpina1e, C1s, Cfd, Plau), cytokine-cytokine receptor interaction (e.g., Ccl11, Amhr2, Lep, Ccl24, Ccl9, Ccl8, Pf4, Fifg, Gm1987, Prl, Ghr, Ccl6), and systemic lupus erythematosus (e.g., C1ra, C7, C3, C4b, Hist2h2ac, H2-eb1, H2-aa, H2-ab1, C1s, Hist2h2aa1) were increased in the *Stat*5NKO group and were reversed by EA treatment. Moreover, the *Stat*5-dependent co-down-regulated genes in the hypothalamus and Epi-WAT were mainly included in the defense response to a high fat diet or a high-nutrition state, and EA treatment could increase this defense ability in obese mice. These regulatory effects of acupuncture on immune balance were similar to the results from other study findings [40,41]. In addition, recent research revealed that group 2 innate lymphoid cells were necessary to the IL-33-induced beiging of white adipose tissue and the limiting of obesity through IL-4 receptor signaling, accompanied with the up-regulation of Ucp1 expression [42]. *Stat*5NKO obese mice in this study were found to have decreased expressions of IL-33 and IL4Ra, which were rescued with EA treatment. Meanwhile, UCP1 expression was increased notably, indicating that the immune cells also contributed to the effect of acupuncture on the browning of white adipose tissue.

Finally, according to the comparison of the RNA-seq data with the list of known genes responsible for monogenic obesity and the verification of qRT-PCR results, we believe that our finding in this study is reliable and of interest, and can provide enough information for further studies to confirm the roles of these DEGs.

## Conclusion

In summary, our data indicate that EA treatment at Zusanli (ST36) and Neiting (ST44) exerts effects of weight-loss on the *Stat*5NKO mice and reverses the obesity related phenotypes by modulating abnormal gene profiles in the hypothalamus and Epi-WAT–especially in the hypothalamus. This study provides, for the first time, informative genome-wide profiles of gene expressions in the *Stat*5NKO obese mice with EA treatment and reveals the potential mechanism of acupuncture on these mice. Our results suggest that acupuncture is an effective approach for treating obesity by directly reducing central pro-adipogenesis signals and regulating abnormal lipid, glucose metabolism, and immune function to maintain homeostasis of energy intake and expenditure.

## Statistical analysis

Data were presented as means ± standard deviation (SD). Statistics analysis was performed using SPSS 18.0; A one-way ANOVA analysis of variance followed by the Tukey HSD test is used for multiple group comparisons. *P* <0.05 was considered statistically significant.

## Supporting information

S1 Fig Deletion efficiency of *Stat*5 in the hypothalamus. STAT5 protein expression was detected by western blot in the hypothalamus, n = 4–5 in each group. 8-week old mice were sacrificed to verify *Stat*5 deletion efficiency. Experiment was repeated 3 times independently. Data were expressed as means ± SD. *P<0.05 vs the *Stat*5fl/fl group.
(PDF)

S2 Fig Schematic diagram of the two acupoints. Zusanli (ST36) is located at the anterior tibia muscle and about 3 mm below the knee joint; Neiting (ST44) is located between the second and third phalanges on the toe.
(DOC)

S3 Fig Representative pictures of Epi-WAT from EA group. (PDF)

S1 Table The primer sequences for qRT-PCR. (DOC)

S2 Table Top 50 *Stat*5 NKO dependent up-regulated differentially expressed genes (DEGs) in hypothalamus. (DOC)

S3 Table Top 50 *Stat*5 NKO dependent up-regulated DEGs in Epi-WAT. (DOC)

S4 Table Top 50 *Stat*5 NKO dependent down-regulated DEGs in hypothalamus. (DOC)

S5 Table Top 50 *Stat*5 NKO dependent down-regulated DEGs in Epi-WAT. (DOC)

S6 Table Top 50 EA dependent down-regulated DEGs in hypothalamus. (DOC)

S7 Table Top 50 EA dependent up-regulated DEGs in hypothalamus. (DOC)

S8 Table Top 50 EA dependent up-regulated DEGs in Epi-WAT. (DOC)

S9 Table Top 50 EA dependent down-regulated DEGs in Epi-WAT. (DOC)
