# Supplementary figures and images for: Genome-wide regulation of electro-acupuncture on the neural Stat5-loss-induced obese mice
Source: PLoS One. 2017 Aug 14;12(8):e0181948. doi: 10.1371/journal.pone.0181948 (PMC5555711; doi:10.1371/journal.pone.0181948)

**S1 Fig.** Deletion efficiency of *Stat5* in the hypothalamus.

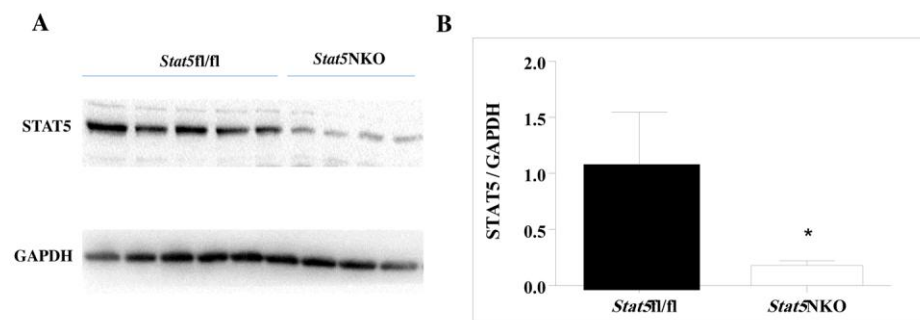

Supplement: S1 Fig — STAT5 protein expression was detected by western blot in the hypothalamus, n = 4–5 in each group. 8-week old mice were sacrificed to verify Stat5 deletion efficiency. Experiment was repeated 3 times independently. Data were expressed as means ± SD. *P<0.05 vs the Stat5fl/fl group. (PDF) [file pone.0181948.s001.pdf]

**S2 Fig.**  Schematic diagram of the two acupoints: Zusanli (ST36 ) and Neiting (ST44).

**
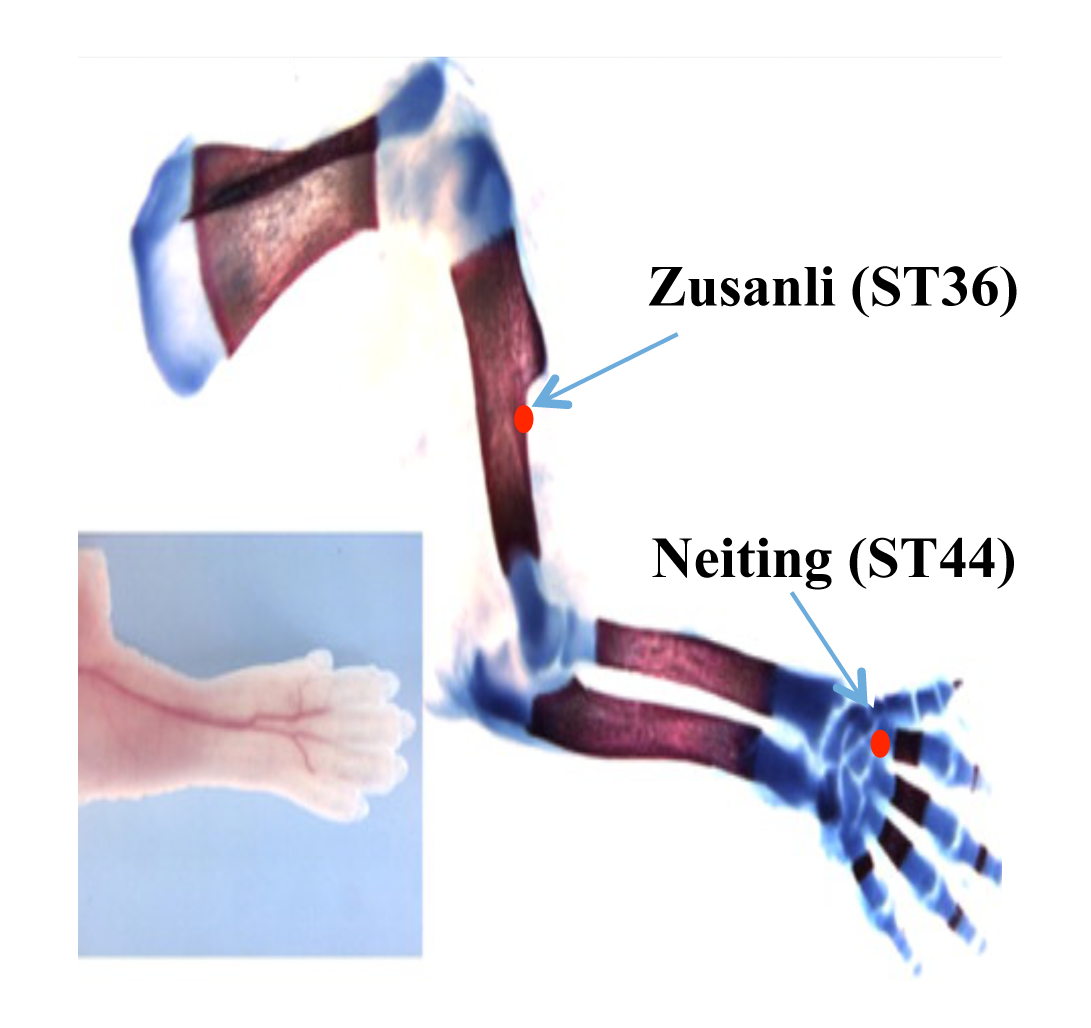
**

Supplement: S2 Fig — Zusanli (ST36) is located at the anterior tibia muscle and about 3 mm below the knee joint; Neiting (ST44) is located between the second and third phalanges on the toe. (DOC) [file pone.0181948.s002.doc]

**S3 Fig.** Representative pictures of Epi-WAT from EA group.

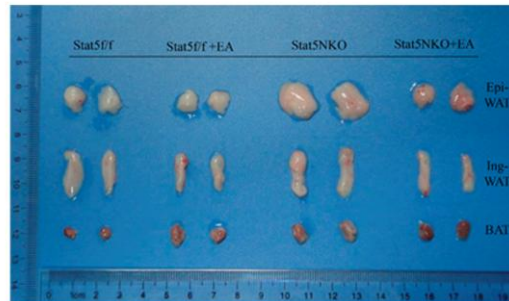

Supplement: S3 Fig — (PDF) [file pone.0181948.s003.pdf]
