# Supplementary material for: Genome-wide regulation of electro-acupuncture on the neural Stat5-loss-induced obese mice
Source: PLoS One. 2017 Aug 14;12(8):e0181948. doi: 10.1371/journal.pone.0181948 (PMC5555711; doi:10.1371/journal.pone.0181948)
Supplement: S1 Table — (DOC) [file pone.0181948.s004.doc]

**S1 Table.** The primer sequences for qRT-PCR.

| Primer | Sequence (5' to 3') |
| --- | --- |
| *Leptin*-F | CATCTGCTGGCCTTCTCCAA |
| *Leptin*-R | TCAAAGCCTCCACCTCTGTG |
| *Pparg*-F | AAGCTGAAACAGCCTCCAAA |
| *Pparg*-R | CAAATTCTTCCCGGTTCTGA |
| *Mc4r*-F | ATCTGTAGCTCCTTGCTCGC |
| *Mc4r*-R | TGCAAGCTGCCCAGATACAA |
| *Ucp*1-F | CACGGGGACCTACAATGCTT |
| *Ucp*1-R | ACAGTAAATGGCAGGGGACG |
| *Sh2b*1-F | AGCTATGTGCCCTCCCAGCG |
| *Sh2b*1-R | CCTGCTCCCGGCCTCACTTCT |
| *Fto*-F | CTCTTGGGACATCGAGACACC |
| *Fto*-R | GGAACTAAACCGAGGCTGTGA |
| *Gnpda*-F | AGCTCCGGCTCTTCGGTCGT |
| *Gnpda*-R | TTGGCTGCCCATTCGCTGGC |
| *Negr*1-F | TGTGACGCAGGAGCACTTCGG |
| *Negr*1-R | AAGAGGTCGCAGGCACTCCC |
| *Gapdh* | GGCACAGTCAAGGCTGAGAATG |
| *Gapdh* | ATGGTGGTGAAGACGCCAGTA |
