# Supplementary material for: Genome-wide regulation of electro-acupuncture on the neural Stat5-loss-induced obese mice
Source: PLoS One. 2017 Aug 14;12(8):e0181948. doi: 10.1371/journal.pone.0181948 (PMC5555711; doi:10.1371/journal.pone.0181948)
Supplement: S2 Table — (DOC) [file pone.0181948.s005.doc]

**S2 Table.** Top 50 *Stat*5 NKO dependent up-regulated differentially expressed genes (DEGs) in hypothalamus.

| Gene name | Description | Hypothalamus | | | Epi-WAT | | |
| --- | --- | --- | --- | --- | --- | --- | --- |
|  |  | fl/fl | NKO | Log2(NKO/fl/fl) | fl/fl | NKO | Log2 (NKO/fl/fl) |
| Gh | growth hormone | 1.27 | 583.76 | 8.84 | 0.01 | 0.54 | 5.58 |
| 1700047G03Rik | RIKEN cDNA 1700047G03 gene | 0.22 | 26.03 | 6.87 | 22.55 | 200.61 | 3.15 |
| Pla2g2e | phospholipase A2, group IIE | 0.03 | 3.52 | 6.79 | 6.09 | 32.34 | 2.41 |
| Mogat2 | monoacylglycerol O-acyltransferase 2 | 0.03 | 3.74 | 6.75 | 1.96 | 30.88 | 3.98 |
| Igfals | insulin-like growth factor binding protein, acid labile subunit | 0.07 | 6.81 | 6.53 | 6.76 | 51.11 | 2.92 |
| Ear11 | ribonuclease, RNase A family, 2A | 0.05 | 5.08 | 6.53 | 7.48 | 30.62 | 2.03 |
| Gm5627 | predicted gene 5627 | 0.05 | 4.19 | 6.25 | 13.39 | 53.78 | 2.01 |
| Krt79 | keratin 79 | 0.01 | 1.01 | 6.19 | 5.57 | 16.42 | 1.56 |
| Plin1 | perilipin 1 | 2.11 | 128.78 | 5.93 | 356.45 | 1227.91 | 1.78 |
| Agpat2 | 1-acylglycerol-3-phosphate O-acyltransferase 2 | 3.94 | 233.31 | 5.89 | 197.19 | 2067.49 | 3.39 |
| 1110059M19Rik | proline rich 32 | 0.06 | 3.07 | 5.72 | 8.36 | 38.49 | 2.20 |
| 2010003K11Rik | RIKEN cDNA 2010003K11 gene | 0.11 | 5.57 | 5.65 | 13.33 | 49.74 | 1.90 |
| Gys2 | glycogen synthase 2 (liver) | 0.04 | 1.86 | 5.64 | 2.96 | 24.14 | 3.03 |
| Prl | prolactin | 0.86 | 40.71 | 5.56 | 0.00 | 0.00 | 0.00 |
| Odf3l1 | outer dense fiber of sperm tails 3-like 1 | 0.08 | 3.88 | 5.52 | 3.75 | 34.32 | 3.19 |
| A530053G22Rik | RIKEN cDNA A530016L24 gene | 0.11 | 5.01 | 5.52 | 22.80 | 78.57 | 1.78 |
| Gm6484 | predicted gene 6484 | 0.19 | 8.15 | 5.44 | 4.64 | 59.95 | 3.69 |
| 9030619P08Rik | lymphocyte antigen 6 complex pseudogene | 0.03 | 1.14 | 5.38 | 4.18 | 9.90 | 1.24 |
| 2610016A17Rik | RIKEN cDNA 2610016A17 gene | 0.20 | 8.33 | 5.37 | 39.72 | 134.24 | 1.76 |
| Mira | mistral long non-coding RNA | 0.04 | 1.57 | 5.37 | 0.00 | 0.00 | 0.00 |
| Lgals1 | lectin, galactoside-binding, soluble, 1 | 20.23 | 833.96 | 5.37 | 2014.20 | 7265.65 | 1.85 |
| Serpina1b,Serpina1d | serine (or cysteine) preptidase inhibitor, clade A, member 1B | 1.27 | 50.93 | 5.33 | 70.04 | 516.50 | 2.88 |
| Ffar2 | free fatty acid receptor 2 | 0.12 | 4.83 | 5.32 | 7.39 | 47.77 | 2.69 |
| Nnmt | nicotinamide N-methyltransferase | 1.53 | 60.19 | 5.30 | 167.17 | 589.68 | 1.82 |
| A530016L24Rik | RIKEN cDNA A530016L24 gene | 0.28 | 10.79 | 5.27 | 32.40 | 112.88 | 1.80 |
| Mrap | melanocortin 2 receptor accessory protein | 2.02 | 77.27 | 5.26 | 266.61 | 700.42 | 1.39 |
| Ccl8 | chemokine (C-C motif) ligand 8 | 0.52 | 19.60 | 5.23 | 68.34 | 133.69 | 0.97 |
| Orm1 | orosomucoid 1 | 2.05 | 73.99 | 5.17 | 235.28 | 756.65 | 1.69 |
| Myl1 | myosin, light polypeptide 1 | 0.20 | 7.20 | 5.16 | 27.10 | 78.41 | 1.53 |
| Pck1 | phosphoenolpyruvate carboxykinase 1, cytosolic | 1.81 | 64.29 | 5.15 | 363.25 | 861.68 | 1.25 |
| Retn | resistin | 20.94 | 701.81 | 5.07 | 3159.80 | 8369.74 | 1.41 |
| Tusc5 | tumor suppressor candidate 5 | 0.69 | 22.73 | 5.05 | 64.61 | 247.72 | 1.94 |
| Retnla | resistin like alpha | 3.85 | 127.42 | 5.05 | 500.26 | 1159.26 | 1.21 |
| Tmem45b | transmembrane protein 45B | 1.78 | 55.32 | 4.96 | 123.44 | 664.58 | 2.43 |
| Cd209d | CD209d antigen | 0.21 | 6.39 | 4.90 | 32.09 | 40.72 | 0.34 |
| Serpina1e | serine (or cysteine) peptidase inhibitor, clade A, member 1E | 0.15 | 4.44 | 4.85 | 70.04 | 516.50 | 2.88 |
| Lpl | lipoprotein lipase | 8.85 | 238.59 | 4.75 | 825.43 | 2877.72 | 1.80 |
| Cd209f | CD209f antigen | 0.15 | 3.71 | 4.61 | 26.79 | 21.27 | -0.33 |
| Adig | adipogenin | 3.13 | 74.09 | 4.56 | 476.11 | 869.88 | 0.87 |
| 1100001G20Rik | WAP four-disulfide core domain 21 | 1.67 | 35.37 | 4.40 | 205.87 | 306.60 | 0.57 |
| O3far1 | omega-3 fatty acid receptor 1 | 0.11 | 2.35 | 4.40 | 8.11 | 27.19 | 1.75 |
| Hoxc8 | homeobox C8 | 0.20 | 4.30 | 4.39 | 26.03 | 62.89 | 1.27 |
| Hp | haptoglobin | 13.32 | 263.64 | 4.31 | 1975.79 | 2749.93 | 0.48 |
| Sucnr1 | succinate receptor 1 | 0.13 | 2.52 | 4.28 | 22.82 | 36.64 | 0.68 |
| Serpina1c | serine (or cysteine) peptidase inhibitor, clade A, member 1C | 0.37 | 7.13 | 4.28 | 70.04 | 516.50 | 2.88 |
| Cidec | cell death-inducing DFFA-like effector c | 21.18 | 408.90 | 4.27 | 2913.34 | 4530.24 | 0.64 |
| Col5a3 | collagen, type V, alpha 3 | 1.31 | 25.01 | 4.26 | 44.19 | 159.82 | 1.85 |
| Cdkn2c | cyclin-dependent kinase inhibitor 2C | 1.48 | 27.85 | 4.24 | 75.12 | 366.63 | 2.29 |
| Lctl | lactase-like | 0.06 | 1.05 | 4.24 | 6.27 | 16.00 | 1.35 |
| Plbd1 | phospholipase B domain containing 1 | 0.38 | 7.06 | 4.22 | 32.87 | 75.47 | 1.20 |
