# Supplementary material for: Genome-wide regulation of electro-acupuncture on the neural Stat5-loss-induced obese mice
Source: PLoS One. 2017 Aug 14;12(8):e0181948. doi: 10.1371/journal.pone.0181948 (PMC5555711; doi:10.1371/journal.pone.0181948)
Supplement: S3 Table — (DOC) [file pone.0181948.s006.doc]

**S3 Table.** Top 50 *Stat*5 NKO dependent up-regulated DEGs in Epi-WAT.

| Gene name | Description | Epi-WAT | | | Hypothalamus | | |
| --- | --- | --- | --- | --- | --- | --- | --- |
|  |  | fl/fl | NKO | Log2(NKO/fl/fl) | fl/fl | NKO | Log2 (NKO/fl/fl) |
| Wdr95 | WD40 repeat domain 95 | 0.00 | 1.95 | +^*^ | 0.22 | 0.36 | 0.74 |
| Gm9696 | predicted gene 9696 | 0.08 | 6.94 | 6.36 | 0.00 | 0.60 | + |
| 2810442I21Rik | Egfr long non-coding downstream RNA | 0.06 | 1.69 | 4.93 | 0.05 | 0.09 | 0.81 |
| Grid1 | glutamate receptor, ionotropic, delta 1 | 0.22 | 5.80 | 4.73 | 22.40 | 13.37 | -0.74 |
| 4930502E18Rik | RIKEN cDNA 4930502E18 gene | 0.06 | 1.33 | 4.53 | 0.45 | 0.34 | -0.41 |
| Fam5b | family with sequence similarity 5, member B | 0.25 | 5.02 | 4.36 | 13.55 | 11.26 | -0.27 |
| Ncan | neurocan | 0.26 | 4.68 | 4.18 | 10.54 | 9.71 | -0.12 |
| Dnmt3l | DNA (cytosine-5-)-methyltransferase 3-like | 0.22 | 3.88 | 4.16 | 0.15 | 0.48 | 1.68 |
| Jakmip3 | janus kinase and microtubule interacting protein 3 | 0.15 | 2.46 | 4.08 | 6.86 | 7.87 | 0.20 |
| Fmr1 | fragile X mental retardation 1 | 8.68 | 142.41 | 4.04 | 6.58 | 20.46 | 1.64 |
| Mogat2 | monoacylglycerol O-acyltransferase 2 | 1.96 | 30.88 | 3.98 | 0.03 | 3.74 | 6.75 |
| Odz4 | odz, odd Oz/ten-m homolog 4 | 4.14 | 58.70 | 3.83 | 8.67 | 7.09 | -0.29 |
| Fgf13 | fibroblast growth factor 13 | 0.75 | 9.95 | 3.74 | 34.84 | 20.68 | -0.75 |
| Gm6484 | predicted gene 6484 | 4.64 | 59.95 | 3.69 | 0.19 | 8.15 | 5.44 |
| Tuba1a | tubulin, alpha 1a | 346.91 | 4387.44 | 3.66 | 910.51 | 1145.76 | 0.33 |
| Rmrp | RNA component of mitochondrial RNAase P | 0.76 | 9.60 | 3.66 | 1.69 | 3.79 | 1.17 |
| Paqr9 | progestin and adipoQ receptor family member | 5.72 | 69.30 | 3.60 | 9.85 | 12.87 | 0.39 |
| Glra1 | glycine receptor, alpha 1 subunit | 1.04 | 12.51 | 3.59 | 4.34 | 1.74 | -1.31 |
| Ucp1 | uncoupling protein 1 | 0.13 | 1.51 | 3.52 | 0.03 | 0.12 | 1.91 |
| Axin2 | axin2 | 0.90 | 10.13 | 3.49 | 5.81 | 7.03 | 0.27 |
| Otop1 | otopetrin 1 | 0.42 | 4.67 | 3.46 | 0.01 | 0.44 | 5.85 |
| Hmgn3 | high mobility group nucleosomal binding | 0.81 | 8.83 | 3.44 | 32.48 | 35.15 | 0.11 |
| Mosc1 | mitochondrial amidoxime reducing | 4.43 | 48.03 | 3.44 | 1.22 | 1.20 | -0.02 |
| Tph2 | tryptophan hydroxylase 2 | 0.57 | 6.16 | 3.43 | 0.23 | 0.62 | 1.45 |
| Agpat2 | 1-acylglycerol-3-phosphate O-acyltransferase 2 | 197.19 | 2067.49 | 3.39 | 3.94 | 233.31 | 5.89 |
| Clstn3 | calsyntenin 3 | 5.63 | 53.09 | 3.24 | 176.25 | 118.88 | -0.57 |
| Rpph1 | ribonuclease P RNA component H1 | 0.65 | 5.98 | 3.20 | 3.28 | 2.87 | -0.19 |
| Odf3l1 | outer dense fiber of sperm tails 3-like 1 | 3.75 | 34.32 | 3.19 | 0.08 | 3.88 | 5.52 |
| Rgs7bp | regulator of G-protein signalling 7 binding protein | 0.59 | 5.27 | 3.16 | 7.79 | 8.73 | 0.16 |
| 1700047G03Rik | RIKEN cDNA 1700047G03 gene | 22.55 | 200.61 | 3.15 | 0.22 | 26.03 | 6.87 |
| Prss27 | protease, serine 27 | 0.62 | 5.47 | 3.14 | 0.03 | 0.55 | 4.27 |
| Dgat2 | diacylglycerol O-acyltransferase 2 | 284.76 | 2431.67 | 3.09 | 17.35 | 250.81 | 3.85 |
| Gys2 | glycogen synthase 2 | 2.96 | 24.14 | 3.03 | 0.04 | 1.86 | 5.64 |
| Unc119 | unc-119 homolog (C. elegans) | 27.75 | 220.39 | 2.99 | 12.38 | 9.11 | -0.44 |
| Lhfpl2 | lipoma HMGIC fusion partner-like 2 | 7.82 | 61.50 | 2.97 | 4.48 | 9.56 | 1.09 |
| Slc22a2 | solute carrier family 22 (organic cation transporter), member 2 | 0.21 | 1.66 | 2.96 | 0.06 | 0.23 | 1.88 |
| Fam20c | family with sequence similarity 20, member C | 8.19 | 63.86 | 2.96 | 22.37 | 22.71 | 0.02 |
| Igfals | insulin-like growth factor binding protein, acid labile subunit | 6.76 | 51.11 | 2.92 | 0.07 | 6.81 | 6.53 |
| Fasn | fatty acid synthase | 180.99 | 1339.11 | 2.89 | 40.38 | 181.69 | 2.17 |
| Serpina1a | serine (or cysteine) peptidase inhibitor, clade A, member 1A | 70.04 | 516.50 | 2.88 | 0.70 | 5.62 | 3.00 |
| Cdkn2a | cyclin-dependent kinase inhibitor 2A | 0.39 | 2.86 | 2.88 | 0.01 | 0.40 | 5.18 |
| Hist2h3c2 | histone cluster 2, H3c2; histone cluster 2 | 2.75 | 19.37 | 2.82 | 2.45 | 3.66 | 0.58 |
| Als2cr12 | amyotrophic lateral sclerosis 2 | 1.83 | 12.81 | 2.81 | 0.23 | 1.73 | 2.91 |
| Bcar3 | breast cancer anti-estrogen resistance 3 | 9.14 | 62.97 | 2.78 | 5.48 | 9.49 | 0.79 |
| Nog | noggin | 0.82 | 5.66 | 2.78 | 5.92 | 5.16 | -0.20 |
| Vac14 | Vac14 homolog | 6.78 | 45.80 | 2.76 | 11.05 | 13.58 | 0.30 |
| Tspan18 | tetraspanin 18 | 3.18 | 20.89 | 2.71 | 3.98 | 3.68 | 0.00 |
| Bpifb6 | BPI fold containing family B, member 6 | 0.24 | 1.53 | 2.69 | 0.02 | 0.21 | 3.24 |
| Ffar2 | free fatty acid receptor 2 | 7.39 | 47.77 | 2.69 | 0.12 | 4.83 | 5.32 |
| Gbp1 | guanylate binding protein 1 | 1.08 | 6.93 | 2.68 | 0.21 | 0.02 | -3.48 |

- “+” represents FPKM value was increased to zero. “-” represents FPKM value was decreased to zero;
