# Supplementary material for: Genome-wide regulation of electro-acupuncture on the neural Stat5-loss-induced obese mice
Source: PLoS One. 2017 Aug 14;12(8):e0181948. doi: 10.1371/journal.pone.0181948 (PMC5555711; doi:10.1371/journal.pone.0181948)
Supplement: S4 Table — (DOC) [file pone.0181948.s007.doc]

**S4 Table.** Top 50 *Stat*5 NKO dependent down-regulated DEGs in hypothalamus.

| Gene name | Description | Hypothalamus | | | Epi-WAT | | |
| --- | --- | --- | --- | --- | --- | --- | --- |
|  |  | fl/fl | NKO | Log2 (NKO/fl/fl) | fl/fl | NKO | Log2 (NKO/fl/fl) |
| Gpx5 | glutathione peroxidase 5 | 19.03 | 0.01 | -10.62 | 0.00 | 0.04 | 0.00 |
| Adam7 | a disintegrin and metallopeptidase domain 7 | 1.93 | 0.01 | -7.66 | 426.64 | 0.02 | -14.55 |
| 9230104L09Rik | RIKEN cDNA 9230104L09 gene | 5.90 | 0.04 | -7.28 | 1105.58 | 0.30 | -11.83 |
| Cst12 | cystatin 12 | 4.94 | 0.05 | -6.61 | 825.34 | 0.00 | - |
| Rnase9 | ribonuclease, RNase A family, 9 (non-active) | 1.36 | 0.02 | -6.28 | 334.42 | 0.00 | - |
| Teddm1 | transmembrane epididymal protein 1 | 2.17 | 0.03 | -6.22 | 422.02 | 0.03 | -13.99 |
| 2210415F13Rik | RIKEN cDNA 2210415F13 gene | 1.22 | 0.04 | -4.89 | 389.58 | 0.50 | -9.61 |
| 3110007F17Rik | RIKEN cDNA 3110007F17 gene | 1.14 | 0.04 | -4.75 | 0.59 | 0.68 | 0.21 |
| Defb30 | defensin beta 30 | 1.28 | 0.06 | -4.35 | 269.14 | 0.15 | -10.77 |
| Defb25 | defensin beta 25 | 10.33 | 0.52 | -4.31 | 2160.17 | 0.38 | -12.48 |
| Spink2 | serine peptidase inhibitor, Kazal type 2 | 1.46 | 0.08 | -4.11 | 260.53 | 0.28 | -9.87 |
| Slc6a3 | solute carrier family 6 (neurotransmitter transporter, dopamine), member 3 | 13.59 | 0.86 | -3.97 | 0.08 | 0.00 | 0.00 |
| Crisp1 | cysteine-rich secretory protein 1 | 1.93 | 0.13 | -3.91 | 257.52 | 5.61 | -5.52 |
| Wfdc10 | WAP four-disulfide core domain 10 | 2.53 | 0.18 | -3.79 | 626.11 | 0.22 | -11.47 |
| Myl2 | myosin, light polypeptide 2, regulatory, cardiac, slow | 2.21 | 0.25 | -3.13 | 0.22 | 0.00 | 0.00 |
| 5830403L16Rik | RIKEN cDNA 5830403L16 gene | 2.13 | 0.26 | -3.06 | 300.26 | 0.02 | -13.83 |
| Mall | mal, T-cell differentiation protein-like | 2.40 | 0.37 | -2.70 | 2.08 | 1.68 | -0.31 |
| Myl10 | myosin, light chain 10, regulatory | 2.07 | 0.34 | -2.59 | 0.50 | 0.00 | - |
| Ly6g5b | lymphocyte antigen 6 complex, locus G5B | 1.45 | 0.28 | -2.36 | 205.77 | 0.28 | -9.53 |
| Mfsd2a | major facilitator superfamily domain containing 2 | 44.49 | 9.40 | -2.24 | 11.28 | 0.60 | -4.24 |
| Cuzd1 | CUB and zona pellucida-like domains 1 | 1.02 | 0.23 | -2.17 | 245.77 | 1.01 | -7.93 |
| Ppp1r2-ps3 | protein phosphatase 1, regulatory (inhibitor) subunit 2, pseudogene 3 | 1.31 | 0.31 | -2.09 | 1.98 | 0.41 | -2.26 |
| Chrna6 | cholinergic receptor, nicotinic, alpha polypeptide 6 | 4.57 | 1.13 | -2.01 | 0.02 | 0.00 | 0.00 |
| Wdr63 | similar to WD repeat domain 63; WD repeat domain 63 | 1.92 | 0.48 | -2.01 | 0.37 | 0.03 | -3.77 |
| Kirrel2 | kin of IRRE like 2 (Drosophila) | 3.79 | 0.95 | -2.00 | 0.04 | 0.00 | 0.00 |
| Lcn2 | lipocalin 2 | 10.40 | 2.72 | -1.93 | 621.74 | 15.40 | -5.34 |
| Chrnb3 | cholinergic receptor, nicotinic, beta polypeptide 3 | 1.10 | 0.31 | -1.83 | 0.01 | 0.00 | 0.00 |
| Hr | hairless | 38.84 | 11.16 | -1.80 | 5.44 | 10.84 | 0.99 |
| Fam107a | family with sequence similarity 107, member A | 162.86 | 48.81 | -1.74 | 3.01 | 0.41 | -2.89 |
| Sgk1 | serum/glucocorticoid regulated kinase 1 | 59.69 | 18.77 | -1.67 | 19.08 | 8.44 | -1.18 |
| Npy | neuropeptide Y | 144.71 | 45.72 | -1.66 | 21.00 | 0.32 | -6.02 |
| Gm8773 | predicted gene 8773 | 1.10 | 0.35 | -1.65 | 0.08 | 0.09 | 0.00 |
| Pglyrp1 | peptidoglycan recognition protein 1 | 18.73 | 6.09 | -1.62 | 1.96 | 1.17 | -0.74 |
| Ly6c2 | lymphocyte antigen 6 complex, locus C2 | 18.54 | 6.09 | -1.61 | 28.00 | 13.31 | -1.07 |
| Edn1 | endothelin 1 | 1.20 | 0.40 | -1.57 | 1.60 | 0.52 | -1.62 |
| Hif3a | hypoxia inducible factor 3, alpha subunit | 2.56 | 0.87 | -1.56 | 1.68 | 0.25 | -2.73 |
| Gm5177 | predicted gene 5177 | 4.62 | 1.58 | -1.55 | 2.12 | 2.30 | 0.12 |
| Pmaip1 | phorbol-12-myristate-13-acetate-induced protein 1 | 1.04 | 0.37 | -1.48 | 0.85 | 0.33 | -1.37 |
| Fkbp5 | FK506 binding protein 5 | 23.88 | 8.63 | -1.47 | 185.86 | 19.80 | -3.23 |
| 2310014L17Rik | RIKEN cDNA 2310014L17 gene | 1.50 | 0.56 | -1.43 | 0.70 | 0.20 | -1.81 |
| Slc10a4 | solute carrier family 10 (sodium/bile acid cotransporter family), member 4 | 5.17 | 1.94 | -1.42 | 0.05 | 0.02 | -1.40 |
| Slc25a34 | solute carrier family 25, member 34 | 6.51 | 2.44 | -1.42 | 0.59 | 0.16 | -1.87 |
| Fzd2 | frizzled homolog 2 (Drosophila) | 7.58 | 2.86 | -1.41 | 1.81 | 2.03 | 0.17 |
| Myoc | myocilin | 9.61 | 3.81 | -1.33 | 0.24 | 0.17 | -0.45 |
| Mt2 | metallothionein 2 | 798.29 | 318.64 | -1.33 | 2781.48 | 268.48 | -3.37 |
| Th | tyrosine hydroxylase | 40.50 | 16.26 | -1.32 | 0.29 | 0.00 | - |
| Srxn1 | sulfiredoxin 1 homolog (S. cerevisiae) | 87.87 | 35.66 | -1.30 | 22.61 | 20.49 | -0.14 |
| Slc39a12 | solute carrier family 39 (zinc transporter), member 12 | 35.56 | 14.69 | -1.28 | 0.25 | 0.05 | -2.25 |
| Slc2a1 | solute carrier family 2 (facilitated glucose transporter), member 1 | 74.72 | 32.19 | -1.21 | 6.67 | 6.52 | -0.03 |
| Acot11 | acyl-CoA thioesterase 11 | 19.52 | 8.50 | -1.20 | 0.53 | 0.49 | -0.11 |
