# Supplementary material for: Genome-wide regulation of electro-acupuncture on the neural Stat5-loss-induced obese mice
Source: PLoS One. 2017 Aug 14;12(8):e0181948. doi: 10.1371/journal.pone.0181948 (PMC5555711; doi:10.1371/journal.pone.0181948)
Supplement: S5 Table — (DOC) [file pone.0181948.s008.doc]

**S5 Table.** Top 50 *Stat*5 NKO dependent down-regulated DEGs in Epi-WAT.

| Gene name | Description | Epi-WAT | | | Hypothalamus | | |
| --- | --- | --- | --- | --- | --- | --- | --- |
|  |  | fl/fl | NKO | Log2(NKO/fl/fl) | fl/fl | NKO | Log2 (NKO/fl/fl) |
| Lcn8 | lipocalin 8 | 1484.72 | 0.03 | -15.67 | 8.25 | 0.00 | - |
| Gm4846 | predicted gene 4846 | 180.36 | 0.00 | -15.38 | 0.92 | 0.00 | - |
| Adam7 | a disintegrin and metallopeptidase domain 7 | 426.64 | 0.02 | -14.55 | 1.93 | 0.01 | -7.66 |
| Defb20 | defensin beta 20 | 1606.96 | 0.09 | -14.06 | 6.45 | 0.00 | - |
| Teddm1 | transmembrane epididymal protein 1 | 422.02 | 0.03 | -13.99 | 2.17 | 0.03 | -6.22 |
| 5830403L16Rik | RIKEN cDNA 5830403L16 gene | 300.26 | 0.02 | -13.83 | 2.13 | 0.26 | -3.06 |
| Defb25 | defensin beta 25 | 2160.17 | 0.38 | -12.48 | 10.33 | 0.52 | -4.31 |
| Spink11 | serine peptidase inhibitor, Kazal type 11 | 195.45 | 0.03 | -12.47 | 0.60 | 0.00 | - |
| Defb45 | defensin beta 45 | 350.85 | 0.07 | -12.34 | 1.43 | 0.00 | - |
| Gm6792 | predicted gene 6792 | 379.79 | 0.08 | -12.27 | 1.30 | 0.03 | -5.35 |
| Lcn6 | lipocalin 6 | 171.97 | 0.04 | -12.24 | 0.34 | 0.00 | - |
| C4bp | complement component 4 binding protein | 136.64 | 0.03 | -12.14 | 0.59 | 0.02 | -4.98 |
| Spink5 | serine peptidase inhibitor, Kazal type 5 | 28.14 | 0.01 | -12.01 | 0.09 | 0.00 | - |
| Gm1110 | predicted gene 1110 | 82.19 | 0.02 | -11.99 | 0.38 | 0.01 | -4.76 |
| 9230104L09Rik | RIKEN cDNA 9230104L09 gene | 1105.58 | 0.30 | -11.83 | 5.90 | 0.04 | -7.28 |
| Ceacam10 | carcinoembryonic antigen-related cell adhesion molecule 10 | 72.02 | 0.02 | -11.67 | 0.29 | 0.06 | -2.26 |
| Rhcg | Rhesus blood group-associated C glycoprotein | 24.24 | 0.01 | -11.60 | 0.60 | 0.78 | 0.39 |
| Wfdc10 | WAP four-disulfide core domain 10 | 626.11 | 0.22 | -11.47 | 2.53 | 0.18 | -3.79 |
| Ros1 | Ros1 proto-oncogene | 3.79 | 0.00 | -11.23 | 0.01 | 0.01 | 0.39 |
| Lipc | lipase, hepatic | 10.51 | 0.00 | -11.06 | 0.02 | 0.02 | -0.21 |
| Wfdc8 | WAP four-disulfide core domain 8 | 14.15 | 0.01 | -10.84 | 0.02 | 0.00 | - |
| Defb30 | defensin beta 30 | 269.14 | 0.15 | -10.77 | 1.28 | 0.06 | -4.35 |
| Rnase13 | ribonuclease, RNase A family, 13 (non-active) | 55.96 | 0.04 | -10.56 | 0.31 | 0.08 | -2.03 |
| Adam28 | a disintegrin and metallopeptidase domain 28 | 106.34 | 0.07 | -10.54 | 0.79 | 0.45 | -0.80 |
| Spink10 | serine peptidase inhibitor, Kazal type 10 | 20.96 | 0.02 | -10.44 | 0.79 | 0.48 | -0.73 |
| Apoa4 | apolipoprotein A-IV | 31.87 | 0.03 | -10.30 | 0.21 | 0.14 | -0.56 |
| Rnase12 | ribonuclease, RNase A family, 12 (non-active) | 152.12 | 0.13 | -10.23 | 0.47 | 0.00 | - |
| Bcl2l15 | BCLl2-like 15 | 44.05 | 0.04 | -10.16 | 0.90 | 1.21 | 0.42 |
| Myo3b | myosin IIIB | 3.27 | 0.00 | -10.04 | 0.33 | 0.23 | -0.53 |
| Rnase10 | ribonuclease, RNase A family, 10 (non-active) | 317.22 | 0.32 | -9.96 | 1.63 | 0.01 | -6.96 |
| Zfp648 | zinc finger protein 648 | 8.05 | 0.01 | -9.90 | 0.18 | 0.14 | -0.35 |
| Spink2 | serine peptidase inhibitor, Kazal type 5 | 260.53 | 0.28 | -9.87 | 1.46 | 0.08 | -4.11 |
| Cryba4 | crystallin, beta A4 | 129.15 | 0.15 | -9.77 | 1.45 | 0.51 | -1.51 |
| 2210415F13Rik | RIKEN cDNA 2210415F13 gene | 389.58 | 0.50 | -9.61 | 1.22 | 0.04 | -4.89 |
| Ly6g5b | lymphocyte antigen 6 complex, locus G5B | 205.77 | 0.28 | -9.53 | 1.45 | 0.28 | -2.36 |
| Gcnt4 | glucosaminyl (N-acetyl) transferase 4, core 2 | 5.91 | 0.01 | -9.52 | 0.35 | 0.37 | 0.07 |
| Cnksr2 | connector enhancer of kinase suppressor of Ras 2 | 2.00 | 0.00 | -9.13 | 4.18 | 2.72 | -0.62 |
| Spinlw1 | serine protease inhibitor-like, with Kunitz and WAP domains 1 (eppin) | 111.95 | 0.24 | -8.89 | 0.34 | 0.03 | -3.66 |
| Bpi | bactericidal permeablility increasing protein | 21.09 | 0.04 | -8.89 | 0.11 | 0.02 | -2.32 |
| Fga | fibrinogen alpha chain | 3.81 | 0.01 | -8.82 | 0.14 | 0.02 | -2.55 |
| Gm5531 | predicted gene 5531 | 61.55 | 0.14 | -8.82 | 0.69 | 0.54 | -0.36 |
| Muc5b | mucin 5, subtype B, tracheobronchial | 3.51 | 0.01 | -8.81 | 0.01 | 0.00 | -1.80 |
| Atp10b | ATPase, class V, type 10B | 4.76 | 0.01 | -8.79 | 0.22 | 0.22 | -0.03 |
| Slc38a5 | solute carrier family 38, member 5 | 223.69 | 0.51 | -8.79 | 2.46 | 2.01 | -0.29 |
| Defb42 | defensin beta 42 | 839.71 | 1.90 | -8.79 | 4.81 | 0.00 | - |
| Slc5a1 | solute carrier family 5 (sodium/glucose cotransporter), member 1 | 1.63 | 0.00 | -8.69 | 0.01 | 0.01 | 0.23 |
| Fut1 | fucosyltransferase 1 | 2.11 | 0.01 | -8.57 | 0.02 | 0.01 | -0.40 |
| Fabp1 | fatty acid binding protein 1, liver | 24.67 | 0.07 | -8.57 | 0.40 | 0.00 | - |
| 2810459M11Rik | RIKEN cDNA 2810459M11 gene | 6.47 | 0.02 | -8.27 | 3.54 | 2.93 | -0.27 |
| Gc | group specific component | 7.57 | 0.02 | -8.27 | 0.14 | 0.00 | - |
