# Supplementary material for: Genome-wide regulation of electro-acupuncture on the neural Stat5-loss-induced obese mice
Source: PLoS One. 2017 Aug 14;12(8):e0181948. doi: 10.1371/journal.pone.0181948 (PMC5555711; doi:10.1371/journal.pone.0181948)
Supplement: S6 Table — (DOC) [file pone.0181948.s009.doc]

**S6 Table.** Top 50 EA dependent down-regulated DEGs in hypothalamus.

| Gene name | Description | FPKM | | | Log2 (fold change) | |
| --- | --- | --- | --- | --- | --- | --- |
|  |  | fl/fl | NKO | EA | NKO vs fl/fl | EA vs NKO |
| Gh | growth hormone | 1.27 | 583.76 | 2.49 | 8.84 | -7.87 |
| Prl | prolactin | 0.86 | 40.71 | 0.69 | 5.56 | -5.88 |
| Cd209d | CD209d antigen | 0.21 | 6.39 | 0.24 | 4.90 | -4.76 |
| 2610016A17Rik | RIKEN cDNA 2610016A17 gene | 0.20 | 8.33 | 0.33 | 5.37 | -4.64 |
| 1110059M19Rik | proline rich 32 | 0.06 | 3.07 | 0.12 | 5.72 | -4.63 |
| Mira | mistral long non-coding RNA | 0.04 | 1.57 | 0.07 | 5.37 | -4.48 |
| Igfals | insulin-like growth factor binding protein, acid labile subunit | 0.07 | 6.81 | 0.31 | 6.53 | -4.47 |
| 1700047G03Rik | RIKEN cDNA 1700047G03 gene | 0.22 | 26.03 | 1.20 | 6.87 | -4.44 |
| Cd209f | CD209f antigen | 0.15 | 3.71 | 0.17 | 4.61 | -4.44 |
| Sucnr1 | succinate receptor 1 | 0.13 | 2.52 | 0.12 | 4.28 | -4.40 |
| Mogat2 | monoacylglycerol O-acyltransferase 2 | 0.03 | 3.74 | 0.18 | 6.75 | -4.34 |
| A530053G22Rik | RIKEN cDNA A530016L24 gene | 0.11 | 5.01 | 0.28 | 5.52 | -4.17 |
| Gm5627 | predicted gene 5627 | 0.05 | 4.19 | 0.24 | 6.25 | -4.15 |
| Plin1 | perilipin 1 | 2.11 | 128.78 | 7.31 | 5.93 | -4.14 |
| Retn | resistin | 20.94 | 701.81 | 40.81 | 5.07 | -4.10 |
| Pla2g2e | phospholipase A2, group IIE | 0.03 | 3.52 | 0.21 | 6.79 | -4.08 |
| 2010003K11Rik | RIKEN cDNA 2010003K11 gene | 0.11 | 5.57 | 0.34 | 5.65 | -4.02 |
| Nnmt | nicotinamide N-methyltransferase | 1.53 | 60.19 | 3.75 | 5.30 | -4.00 |
| 1100001G20Rik | WAP four-disulfide core domain 21 | 1.67 | 35.37 | 2.21 | 4.40 | -4.00 |
| Ear11 | ribonuclease, RNase A family, 2A | 0.05 | 5.08 | 0.32 | 6.53 | -3.97 |
| Cd209g | CD209g antigen | 0.21 | 3.52 | 0.22 | 4.90 | -3.97 |
| Mrap | melanocortin 2 receptor accessory protein | 2.02 | 77.27 | 4.94 | 5.26 | -3.97 |
| Lgals1 | lectin, galactoside-binding, soluble, 1 | 20.23 | 833.96 | 53.36 | 5.37 | -3.97 |
| Agpat2 | 1-acylglycerol-3-phosphate O-acyltransferase 2 | 3.94 | 233.31 | 14.95 | 5.89 | -3.96 |
| O3far1 | omega-3 fatty acid receptor 1 | 0.11 | 2.35 | 0.15 | 4.40 | -3.96 |
| Tusc5 | tumor suppressor candidate 5 | 0.69 | 22.73 | 1.50 | 5.05 | -3.93 |
| Lctl | lactase-like | 0.06 | 1.05 | 0.07 | 4.24 | -3.92 |
| A530016L24Rik | RIKEN cDNA A530016L24 gene | 0.28 | 10.79 | 0.73 | 5.27 | -3.89 |
| Lpl | lipoprotein lipase | 8.85 | 238.59 | 16.20 | 4.75 | -3.88 |
| Myl1 | myosin, light polypeptide 1 | 0.20 | 7.20 | 0.49 | 5.16 | -3.87 |
| Pck1 | phosphoenolpyruvate carboxykinase 1, cytosolic | 1.81 | 64.29 | 4.47 | 5.15 | -3.85 |
| Hoxc8 | homeobox C8 | 0.20 | 4.30 | 0.30 | 4.39 | -3.84 |
| Orm1,Orm3 | orosomucoid 1 | 2.05 | 73.99 | 5.22 | 5.17 | -3.82 |
| Adig | adipogenin | 3.13 | 74.09 | 5.25 | 4.56 | -3.82 |
| Retnla | resistin like alpha | 3.85 | 127.42 | 9.05 | 5.05 | -3.82 |
| Hp | haptoglobin | 13.32 | 263.64 | 18.98 | 4.31 | -3.80 |
| Cdkn2c | cyclin-dependent kinase inhibitor 2C | 1.48 | 27.85 | 2.01 | 4.24 | -3.79 |
| Mmp7 | matrix metallopeptidase 7 | 0.16 | 1.05 | 0.08 | 2.69 | -3.77 |
| Tmem45b | transmembrane protein 45B | 1.78 | 55.32 | 4.10 | 4.96 | -3.76 |
| Cidec | cell death-inducing DFFA-like effector c | 21.18 | 408.90 | 30.31 | 4.27 | -3.75 |
| Serpina1b | serine (or cysteine) preptidase inhibitor, clade A, member 1B | 1.27 | 50.93 | 3.82 | 5.33 | -3.74 |
| Aoc3 | amine oxidase, copper containing 3 | 2.00 | 35.41 | 2.67 | 4.14 | -3.73 |
| Dmrt2 | doublesex and mab-3 related transcription factor 2 | 0.28 | 3.94 | 0.30 | 3.83 | -3.72 |
| Ffar2 | free fatty acid receptor 2 | 0.12 | 4.83 | 0.37 | 5.32 | -3.70 |
| Car3 | carbonic anhydrase III, muscle specific | 112.42 | 1479.78 | 114.21 | 3.72 | -3.70 |
| Acp5 | acid phosphatase 5, tartrate resistant | 1.98 | 28.87 | 2.29 | 3.87 | -3.65 |
| Lep | leptin | 4.33 | 58.59 | 4.66 | 3.76 | -3.65 |
| Ccl8 | chemokine (C-C motif) ligand 8 | 0.52 | 19.60 | 1.57 | 5.23 | -3.65 |
| Gm11428 | WAP four-disulfide core domain 17 | 1.20 | 19.66 | 1.60 | 4.04 | -3.62 |
| Col3a1 | collagen, type III, alpha 1 | 1.12 | 18.01 | 1.47 | 4.01 | -3.62 |
