# Supplementary material for: Genome-wide regulation of electro-acupuncture on the neural Stat5-loss-induced obese mice
Source: PLoS One. 2017 Aug 14;12(8):e0181948. doi: 10.1371/journal.pone.0181948 (PMC5555711; doi:10.1371/journal.pone.0181948)
Supplement: S7 Table — (DOC) [file pone.0181948.s010.doc]

**S7 Table.** Top 50 EA dependent up-regulated DEGs in hypothalamus.

| Gene name | Description | FPKM | | | Log2 (fold change) | |
| --- | --- | --- | --- | --- | --- | --- |
|  |  | fl/fl | NKO | EA | NKO vs fl/fl | EA vs NKO |
| AY761185 | cDNA sequence AY761185; similar to CRS4C-6 | 2.31 | 0.00 | 1.17 | - | + |
| Cst11 | cystatin 11 | 3.11 | 0.00 | 1.55 | - | + |
| Defb20 | defensin beta 20 | 6.45 | 0.00 | 3.19 | - | + |
| Defb28 | defensin beta 28 | 1.07 | 0.00 | 1.28 | - | + |
| Defb48 | defensin beta 48 | 7.00 | 0.00 | 2.99 | - | + |
| Lcn8 | lipocalin 8 | 8.25 | 0.00 | 3.71 | - | + |
| Lcn9 | lipocalin 9 | 5.65 | 0.00 | 2.88 | - | + |
| Mir680-2 | microRNA 680-2 | 28.99 | 0.00 | 49.46 | - | + |
| S100a5 | S100 calcium binding protein A5 | 0.04 | 0.00 | 3.53 | - | + |
| Spag11b | sperm associated antigen 11B | 12.93 | 0.00 | 5.70 | - | + |
| Gpx5 | glutathione peroxidase 5 | 19.03 | 0.01 | 8.98 | -10.62 | 9.53 |
| 9230104L09Rik | RIKEN cDNA 9230104L09 gene | 5.90 | 0.04 | 2.16 | -7.28 | 5.83 |
| Cst12 | cystatin 12 | 4.94 | 0.05 | 1.71 | -6.61 | 5.08 |
| Ntrk1 | neurotrophic tyrosine kinase, receptor, type 1 | 0.77 | 0.32 | 1.08 | -1.27 | 3.14 |
| Defb25 | defensin beta 25 | 10.33 | 0.52 | 5.32 | -4.31 | 2.68 |
| Crisp1 | cysteine-rich secretory protein 1 | 1.93 | 0.13 | 1.13 | -3.91 | 2.47 |
| Wfdc10 | WAP four-disulfide core domain 10 | 2.53 | 0.18 | 1.52 | -3.79 | 3.06 |
| Pmaip1 | phorbol-12-myristate-13-acetate-induced protein 1 | 1.04 | 0.37 | 2.39 | -1.48 | 2.25 |
| Samd3 | sterile alpha motif domain containing 3 | 0.57 | 0.21 | 1.26 | -1.43 | 2.32 |
| Slc6a3 | solute carrier family 6 (neurotransmitter transporter), member 3 | 13.59 | 0.86 | 4.80 | -3.97 | 2.47 |
| Chat | choline acetyltransferase | 0.75 | 0.23 | 1.23 | -1.68 | 2.38 |
| Lhx8 | LIM homeobox protein 8 | 0.96 | 0.39 | 1.95 | -1.31 | 2.32 |
| Ttr | transthyretin | 1.76 | 2.07 | 9.87 | -0.23 | 2.25 |
| Edn1 | endothelin 1 | 1.20 | 0.40 | 1.64 | -1.57 | 2.02 |
| Alb | albumin | 1.33 | 0.44 | 1.64 | -1.61 | 1.91 |
| Wdr63 | WD repeat domain 63 | 1.92 | 0.48 | 1.76 | -2.01 | 1.88 |
| Fpr2 | ormyl peptide receptor 2 | 0.90 | 0.57 | 1.79 | -0.66 | 1.65 |
| Gm694 | predicted gene 694 | 2.61 | 0.86 | 2.55 | -1.60 | 1.56 |
| Foxg1 | forkhead box G1 | 1.85 | 1.55 | 4.51 | -0.25 | 1.54 |
| Tbr1 | T-box brain gene 1 | 0.53 | 0.44 | 1.19 | -0.27 | 1.44 |
| Lhx6 | LIM homeobox protein 6 | 3.28 | 1.85 | 4.82 | -0.83 | 1.39 |
| Pglyrp1 | peptidoglycan recognition protein 1 | 18.73 | 6.09 | 15.05 | -1.62 | 1.31 |
| Mfsd2a | major facilitator superfamily domain containing 2A | 44.49 | 9.40 | 22.91 | -2.24 | 1.28 |
| Nts | neurotensin | 34.52 | 22.47 | 48.71 | -0.62 | 1.12 |
| Fkbp5 | FK506 binding protein 5 | 23.88 | 8.63 | 17.64 | -1.47 | 1.03 |
