# Supplementary material for: Genome-wide regulation of electro-acupuncture on the neural Stat5-loss-induced obese mice
Source: PLoS One. 2017 Aug 14;12(8):e0181948. doi: 10.1371/journal.pone.0181948 (PMC5555711; doi:10.1371/journal.pone.0181948)
Supplement: S8 Table — (DOC) [file pone.0181948.s011.doc]

**S8 Table.** Top 50 EA dependent up-regulated DEGs in Epi-WAT.

| Gene name | Description | FPKM | | | Log2 (fold change) | |
| --- | --- | --- | --- | --- | --- | --- |
|  |  | fl/fl | NKO | EA | NKO vs fl/fl | EA vs NKO |
| Ceacam10 | carcinoembryonic antigen-related cell adhesion molecule 10 | 72.02 | 0.02 | 5.41 | -11.88 | 8.15 |
| Ucp1 | uncoupling protein 1 | 0.13 | 1.30 | 257.39 | 3.31 | 7.62 |
| Wfdc10 | WAP four-disulfide core domain 10 | 626.11 | 0.19 | 34.07 | -11.69 | 7.49 |
| Slfnl1 | schlafen like 1 | 0.64 | 0.01 | 1.30 | -6.42 | 7.45 |
| Sost | sclerostin | 0.03 | 0.02 | 2.44 | -0.57 | 7.14 |
| Mup8 | major urinary protein 8 | 0.01 | 0.06 | 8.90 | 3.23 | 7.10 |
| Gpx5 | glutathione peroxidase 5 | 0.00 | 0.04 | 4.39 | 0.00 | 7.02 |
| Mup13 | major urinary protein 13 | 0.11 | 0.09 | 11.65 | -0.31 | 7.01 |
| Bglap-rs1 | bone gamma-carboxyglutamate protein 3 | 8.74 | 0.07 | 9.06 | -6.94 | 6.99 |
| Mup15 | major urinary protein 15 | 0.15 | 0.28 | 35.03 | 0.90 | 6.97 |
| Mup12 | major urinary protein 12 | 0.01 | 0.09 | 11.32 | 3.04 | 6.92 |
| Mup14 | major urinary protein 14 | 0.05 | 0.29 | 34.29 | 2.47 | 6.90 |
| Mup1 | major urinary protein 1 | 0.14 | 0.10 | 11.62 | -0.48 | 6.83 |
| Cdh17 | cadherin 17 | 1.29 | 0.02 | 2.00 | -6.09 | 6.72 |
| 1700090G07Rik | transmembrane protein 247 | 0.84 | 0.02 | 2.17 | -5.35 | 6.71 |
| Fabp3 | fatty acid binding protein 3, muscle and heart | 3.42 | 1.38 | 142.96 | -1.31 | 6.69 |
| Cwh43 | cell wall biogenesis 43 C-terminal homolog | 1.81 | 0.06 | 6.45 | -4.82 | 6.65 |
| LOC100048884 | novel member of the major urinary protein (Mup) gene family | 0.01 | 0.19 | 18.57 | 3.91 | 6.63 |
| Fxyd4 | FXYD domain containing ion transport regulator 4 | 0.64 | 0.59 | 57.24 | -0.12 | 6.59 |
| Crygb | crystallin, gamma B | 0.05 | 0.06 | 6.04 | 0.44 | 6.59 |
| Gm10639 | predicted gene 10639 | 0.14 | 0.01 | 1.09 | -3.65 | 6.59 |
| Elf5 | E74-like factor 5 | 0.90 | 0.03 | 2.56 | -5.08 | 6.59 |
| Gm5087 | predicted gene 5087 | 0.04 | 0.04 | 3.43 | -0.13 | 6.59 |
| Mup19 | major urinary protein 19 | 0.01 | 0.22 | 20.55 | 3.99 | 6.56 |
| Adprhl1 | ADP-ribosylhydrolase like 1 | 0.02 | 0.05 | 4.49 | 1.60 | 6.54 |
| Crisp1 | cysteine-rich secretory protein 1 | 257.52 | 4.86 | 451.50 | -5.73 | 6.54 |
| 1700029F12Rik | RIKEN cDNA 1700029F12 gene | 3.31 | 0.02 | 2.22 | -7.09 | 6.52 |
| Akr1b7 | aldo-keto reductase family 1, member B7 | 65.72 | 60.91 | 5497.93 | -0.11 | 6.50 |
| Ptgs2 | prostaglandin-endoperoxide synthase 2 | 1.02 | 0.90 | 80.35 | -0.19 | 6.48 |
| 1700080E11Rik | RIKEN cDNA 1700080E11 gene | 0.74 | 0.02 | 1.64 | -5.29 | 6.44 |
| Serpina1f | serine (or cysteine) peptidase inhibitor, clade A, member 1F | 51.82 | 1.09 | 91.24 | -5.57 | 6.38 |
| Gm9573 | predicted gene 9573 | 0.04 | 0.09 | 7.15 | 1.04 | 6.37 |
| Prm1 | protamine 1 | 29.97 | 1.71 | 141.11 | -4.13 | 6.37 |
| Anxa13 | annexin A13 | 1.89 | 0.18 | 15.03 | -3.37 | 6.36 |
| Bcl2l15 | BCLl2-like 15 | 44.05 | 0.03 | 2.05 | -10.78 | 6.35 |
| Irx1 | Iroquois related homeobox 1 | 0.77 | 0.13 | 10.70 | -2.56 | 6.35 |
| 7530420F21Rik | prostaglandin-endoperoxide synthase 2, opposite strand | 0.03 | 0.04 | 3.19 | 0.58 | 6.33 |
| Hils1 | histone linker H1 domain, spermatid-specific 1 | 1.47 | 0.04 | 2.93 | -5.33 | 6.32 |
| Pax2 | paired box 2 | 1.69 | 0.02 | 1.82 | -6.20 | 6.30 |
| Acbd7 | acyl-CoA binding domain containing 7 | 9.53 | 0.13 | 10.43 | -6.17 | 6.30 |
| Gm2083 | major urinary protein LOC100048885 | 0.05 | 0.98 | 75.75 | 4.16 | 6.28 |
| Crygc | crystallin, gamma C | 0.77 | 1.87 | 144.90 | 1.28 | 6.27 |
| Spink8 | serine peptidase inhibitor, Kazal type 8 | 704.27 | 3.67 | 278.09 | -7.59 | 6.25 |
| Mup16 | major urinary protein 16 | 0.03 | 0.17 | 12.35 | 2.65 | 6.21 |
| Ankrd22 | ankyrin repeat domain 22 | 0.88 | 0.02 | 1.34 | -5.60 | 6.19 |
| Ropn1 | ropporin, rhophilin associated protein 1 | 1.31 | 0.02 | 1.27 | -6.21 | 6.16 |
| Aqp2 | aquaporin 2 | 2.14 | 2.66 | 190.11 | 0.31 | 6.16 |
| Wfdc15b | WAP four-disulfide core domain 15B | 74.48 | 0.67 | 47.18 | -6.80 | 6.14 |
| Tgm3 | transglutaminase 3 | 0.31 | 0.04 | 2.81 | -2.93 | 6.13 |
| Defb42 | defensin beta 42 | 839.71 | 1.66 | 115.01 | -8.98 | 6.12 |
