# Supplementary material for: Genome-wide regulation of electro-acupuncture on the neural Stat5-loss-induced obese mice
Source: PLoS One. 2017 Aug 14;12(8):e0181948. doi: 10.1371/journal.pone.0181948 (PMC5555711; doi:10.1371/journal.pone.0181948)
Supplement: S9 Table — (DOC) [file pone.0181948.s012.doc]

**S9 Table.** Top 50 EA dependent down-regulated DEGs in Epi-WAT.

| Gene name | Description | FPKM | | | Log2 (fold change) | |
| --- | --- | --- | --- | --- | --- | --- |
|  |  | fl/fl | NKO | EA | NKO vs fl/fl | EA vs NKO |
| Gbp1 | guanylate binding protein 1, interferon-inducible | 1.08 | 5.97 | 0.13 | 2.46 | -5.54 |
| Crabp1 | cellular retinoic acid binding protein I | 1.72 | 1.55 | 0.04 | -0.15 | -5.27 |
| Grid1 | glutamate receptor, ionotropic, delta 1 | 0.22 | 4.97 | 0.15 | 4.50 | -5.07 |
| Mmp7 | matrix metallopeptidase 7 | 30.31 | 3.52 | 0.11 | -3.11 | -4.97 |
| Fam5b | family with sequence similarity 5, member B | 0.25 | 4.30 | 0.14 | 4.13 | -4.93 |
| 1700047G03Rik | RIKEN cDNA 1700047G03 gene | 22.55 | 173.78 | 7.27 | 2.95 | -4.58 |
| Bpifb6 | BPI fold containing family B, member 6 | 0.24 | 1.31 | 0.06 | 2.47 | -4.40 |
| Pla2g2e | phospholipase A2, group IIE | 6.09 | 27.59 | 1.68 | 2.18 | -4.04 |
| Mt3 | metallothionein 3 | 11.95 | 9.08 | 0.70 | -0.40 | -3.70 |
| Serpina1a | serine (or cysteine) preptidase inhibitor, clade A, member 1A | 70.04 | 434.11 | 35.24 | 2.63 | -3.62 |
| Tuba1a | predicted gene 7172; similar to tubulin, alpha 1; tubulin, alpha 1A | 346.91 | 3758.31 | 325.53 | 3.44 | -3.53 |
| 2810442I21Rik | RIKEN cDNA 2810442I21 gene | 0.06 | 1.47 | 0.13 | 4.73 | -3.53 |
| Hmgn3 | high mobility group nucleosomal binding domain 3 | 0.81 | 7.75 | 0.69 | 3.25 | -3.49 |
| Dnmt3l | similar to DNA cytosine-5 methyltransferase 3-like protein | 0.22 | 3.33 | 0.30 | 3.94 | -3.47 |
| Lhfpl2 | lipoma HMGIC fusion partner-like 2 | 7.82 | 53.40 | 5.08 | 2.77 | -3.39 |
| Lrrc4c | leucine rich repeat containing 4C | 1.42 | 3.45 | 0.33 | 1.28 | -3.39 |
| Hist2h3b | histone cluster 2, H3b | 1.85 | 9.42 | 0.99 | 2.35 | -3.25 |
| Hist1h1d | histone cluster 1, H1d | 0.24 | 1.24 | 0.13 | 2.36 | -3.22 |
| Tph2 | tryptophan hydroxylase 2 | 0.57 | 5.39 | 0.60 | 3.24 | -3.17 |
| Cpa2 | carboxypeptidase A2, pancreatic | 1.93 | 4.09 | 0.46 | 1.08 | -3.17 |
| Ear11 | eosinophil-associated, ribonuclease A family, member 11 | 7.48 | 26.76 | 3.00 | 1.84 | -3.16 |
| Retnla | resistin like alpha | 500.26 | 999.93 | 115.20 | 1.00 | -3.12 |
| Fmr1 | fragile X mental retardation syndrome 1 homolog | 8.68 | 124.40 | 15.21 | 3.84 | -3.03 |
| Adh4 | alcohol dehydrogenase 4 (class II), pi polypeptide | 0.35 | 1.40 | 0.17 | 2.01 | -3.03 |
| Kcnj15 | potassium inwardly-rectifying channel, subfamily J, member 15 | 4.67 | 11.16 | 1.41 | 1.26 | -2.98 |
| 1500017E21Rik | RIKEN cDNA 1500017E21 gene | 0.70 | 1.64 | 0.21 | 1.23 | -2.98 |
| Odf3l1 | outer dense fiber of sperm tails 3-like 1 | 3.75 | 29.48 | 3.84 | 2.97 | -2.94 |
| Ptch2 | patched homolog 2 | 1.77 | 4.33 | 0.59 | 1.29 | -2.89 |
| Dfna5 | deafness, autosomal dominant 5 (human) | 2.87 | 12.79 | 1.75 | 2.15 | -2.87 |
| Fam20c | family with sequence similarity 20, member C | 8.19 | 54.51 | 7.50 | 2.73 | -2.86 |
| Mrgprg | MAS-related GPR, member G | 0.49 | 2.41 | 0.35 | 2.29 | -2.80 |
| Nog | noggin | 0.82 | 4.84 | 0.78 | 2.56 | -2.63 |
| Mogat2 | monoacylglycerol O-acyltransferase 2 | 1.96 | 26.46 | 4.29 | 3.75 | -2.63 |
| Folh1 | folate hydrolase | 2.85 | 4.04 | 0.66 | 0.51 | -2.61 |
| Tfpi2 | tissue factor pathway inhibitor 2 | 0.71 | 2.11 | 0.35 | 1.57 | -2.61 |
| Gm6682 | tubulin, alpha 1C; predicted gene 6682 | 61.86 | 251.45 | 41.74 | 2.02 | -2.59 |
| Lrrc39 | leucine rich repeat containing 39 | 6.85 | 14.48 | 2.45 | 1.08 | -2.57 |
| 9030625A04Rik | RIKEN cDNA 9030625A04 gene | 2.21 | 8.65 | 1.48 | 1.97 | -2.55 |
| Bcar3 | breast cancer anti-estrogen resistance 3 | 9.14 | 53.99 | 9.24 | 2.56 | -2.55 |
| Morc4 | microrchidia 4 | 3.95 | 12.76 | 2.21 | 1.69 | -2.53 |
| Plagl1 | pleiomorphic adenoma gene-like 1 | 4.05 | 15.11 | 2.62 | 1.90 | -2.53 |
| Col5a3 | collagen, type V, alpha 3 | 44.19 | 135.76 | 23.68 | 1.62 | -2.52 |
| Hdac9 | histone deacetylase 9 | 0.69 | 3.82 | 0.67 | 2.48 | -2.51 |
| Sc5d | sterol-C5-desaturase (fungal ERG3, delta-5-desaturase) homolog | 13.35 | 33.95 | 5.98 | 1.35 | -2.50 |
| Tmem182 | transmembrane protein 182 | 18.60 | 62.11 | 10.95 | 1.74 | -2.50 |
| Ffar2 | free fatty acid receptor 2 | 7.39 | 41.23 | 7.29 | 2.48 | -2.50 |
| Rrad | Ras-related associated with diabetes | 2.77 | 11.46 | 2.03 | 2.05 | -2.49 |
| Prr16 | proline rich 16 | 4.20 | 10.21 | 1.81 | 1.28 | -2.49 |
| Rbm28 | RNA binding motif protein 28 | 45.13 | 85.11 | 15.19 | 0.92 | -2.49 |
| C6 | complement component 6 | 4.52 | 13.26 | 2.37 | 1.55 | -2.48 |
